# Identification of Trigeminal Sensory Neuronal Types Innervating Masseter Muscle

**DOI:** 10.1523/ENEURO.0176-21.2021

**Published:** 2021-10-12

**Authors:** Karen A. Lindquist, Sergei Belugin, Anahit H. Hovhannisyan, Tatiana M. Corey, Adam Salmon, Armen N. Akopian

**Affiliations:** 1 Integrated Biomedical Sciences (IBMS) Program, The University of Texas Health Science Center at San Antonio, San Antonio, TX 78229; 2 Endodontics, The University of Texas Health Science Center at San Antonio, San Antonio, TX 78229; 3 Pharmacology, The University of Texas Health Science Center at San Antonio, San Antonio, TX 78229; 4 Molecular Medicine, The University of Texas Health Science Center at San Antonio, San Antonio, TX 78229; 5 Laboratory Animal Resources Departments, The University of Texas Health Science Center at San Antonio, San Antonio, TX 78229; 6 Sam and Ann Barshop Institute for Longevity and Aging Studies, The University of Texas Health Science Center at San Antonio, San Antonio, TX 78229; 7 South Texas Veterans Health Care System, Geriatric Research Education and Clinical Center San Antonio, TX 78229

**Keywords:** masseter muscle, myogenous temporomandibular disorders, orofacial pain, sensory neurons, trigeminal

## Abstract

Understanding masseter muscle (MM) innervation is critical for the study of cell-specific mechanisms of pain induced by temporomandibular disorder (TMDs) or after facial surgery. Here, we identified trigeminal (TG) sensory neuronal subtypes (MM TG neurons) innervating MM fibers, masseteric fascia, tendons, and adjusted tissues. A combination of patch clamp electrophysiology and immunohistochemistry (IHC) on TG neurons back-traced from reporter mouse MM found nine distinct subtypes of MM TG neurons. Of these neurons, 24% belonged to non-peptidergic IB-4^+^/TRPA1^–^ or IB-4^+^/TRPA1^+^ groups, while two TRPV1^+^ small-sized neuronal groups were classified as peptidergic/CGRP^+^. One small-sized CGRP^+^ neuronal group had a unique electrophysiological profile and were recorded from Nav1.8^–^ or trkC^+^ neurons. The remaining CGRP^+^ neurons were medium-sized, could be divided into Nav1.8^–^/trkC^–^ and Nav1.8^low^/trkC^+^ clusters, and showed large 5HT-induced current. The final two MM TG neuronal groups were trkC^+^ and had no Nav1.8 and CGRP. Among MM TG neurons, TRPV1^+^/CGRP^–^ (somatostatin^+^), tyrosine hydroxylase (TH)^+^ (C-LTMR), TRPM8^+^, MrgprA3^+^, or trkB^+^ (Aδ-LTMR) subtypes have not been detected. Masseteric muscle fibers, tendons and masseteric fascia in mice and the common marmoset, a new world monkey, were exclusively innervated by either CGRP^+^/NFH^+^ or CGRP^–^/NFH^+^ medium-to-large neurons, which we found using a Nav1.8-YFP reporter, and labeling with CGRP, TRPV1, neurofilament heavy chain (NFH) and pgp9.5 antibodies. These nerves were mainly distributed in tendon and at junctions of deep-middle-superficial parts of MM. Overall, the data presented here demonstrates that MM is innervated by a distinct subset of TG neurons, which have unique characteristics and innervation patterns.

## Significance Statement

Identification of sensory neuron subtypes innervating masseter muscle (MM) will enable the study of cell-specific mechanisms of masticatory myofascial pain, including temporomandibular disorder (TMD) and after restorative surgeries involving MM. Combining back tracing from MM, patch-clamp electrophysiology, and immunohistochemistry (IHC) with sensory neuronal markers on mouse and nonhuman primate tissues, we identified trigeminal (TG) neuronal groups innervating MM (MM TG neurons). MM and adjacent tissues are innervated by 9 distinct types of TG neurons, some of which are substantially different from L3–L5 DRG neurons. However, MM fibers, tendon, muscle-tendon junction, and fascia in mice and common marmosets are exclusively innervated by medium-to-large neurons. These neurofilament heavy chain (NFH)-positive sensory nerve fibers are mainly distributed in tendon and at junctions between deep, middle, and superficial parts of MM.

## Introduction

Myofascial pain is widely prevalent in the general population (Cimmino et al., 2011). Pain related to mastication muscles, which are comprised of the temporalis, medial pterygoid, lateral pterygoid, and masseter muscle (MM), is referred to as masticatory myofascial pain. Masticatory myofascial pain may manifest as facial pain, headache, soreness, and fatigue of the masticatory muscles (Gerwin, 2001). This pain type is detected among patients with temporomandibular disorders (TMDs; Lobbezoo et al., 1996; Slade et al., 2016), myofascial pain syndrome (Desai et al., 2013; Galasso et al., 2020), and after cranio-facial surgeries done to repair facial paralysis, facelift procedures, or tissue trauma affecting MM (Wieckiewicz et al., 2015; Lassus et al., 2018).

**Figure 13.**
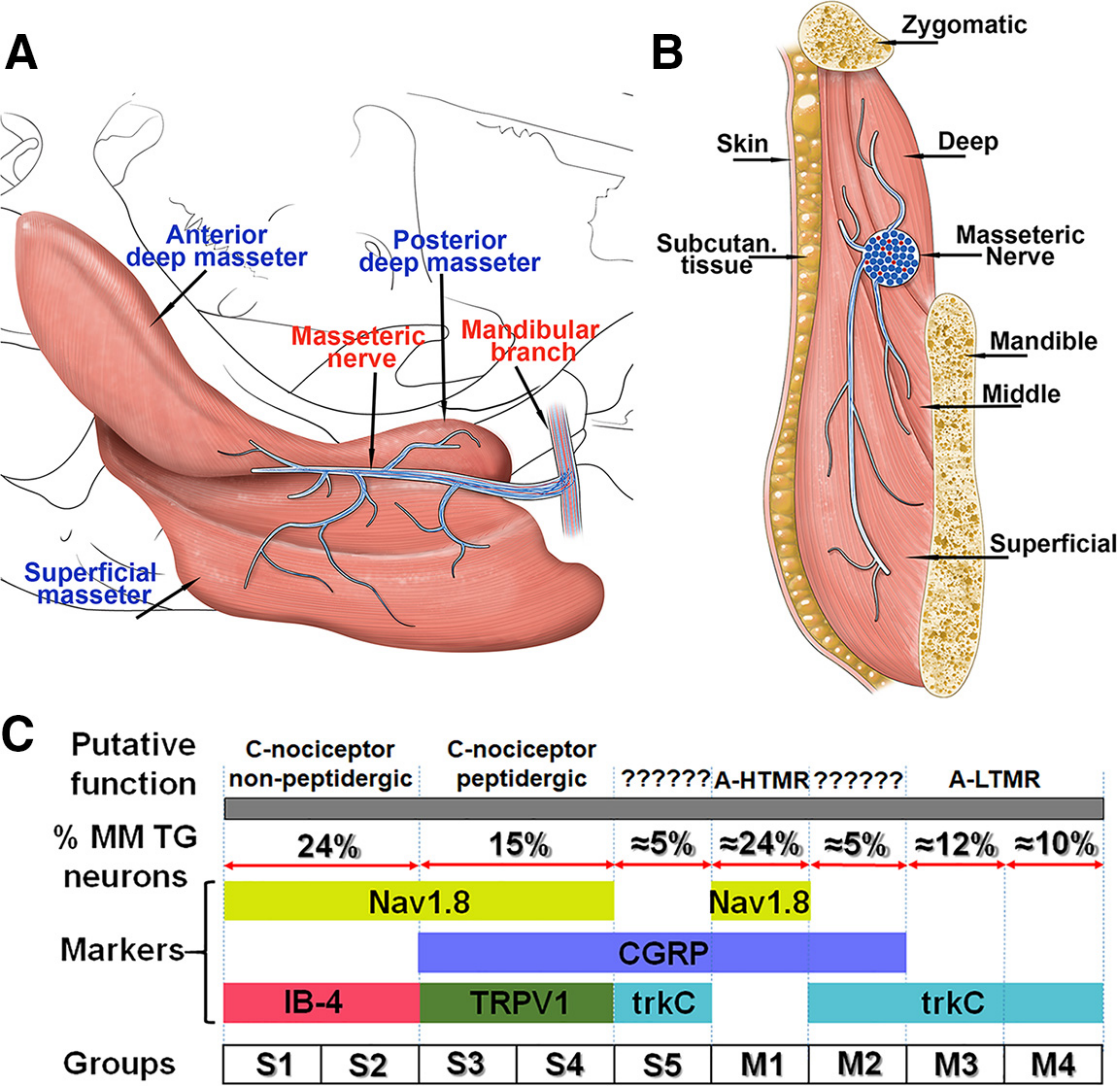
Schematic abstract of results. ***A***, Schematic of different parts of masseteric muscle (labeled) and branches of masseteric nerve (labeled). ***B***, Schematic of coronal section of MM with adjusted tissues. These adjacent tissues and MM components are labeled. On the panels ***A***, ***B***, blue lines and dots mark A-fibers, and red lines and dots represent C-fibers in mandibular and masseteric nerves and in their branches. ***C***, Schematic TG neuronal groups (S1–S5, M1–M4) innervating MM. Expressions of neuronal markers are specified. Percentage of WGA^+^ for each neuronal group based on IHC is shown. Putative functions of MM TG neuronal groups are suggested. A-HTMR are medium-sized A-nociceptors or high threshold mechanoreceptors. A-LTMR are medium-to-large-sized A-low threshold mechanoreceptors. Putative functions of S5 and M2 are ambiguous, do not map onto previously described classes of DRG neurons, and marked as “unknown.”

MM consists of three heads (superficial, middle, and deep) of muscle fibers, masseteric fascia, and tendons; and is innervated by the masseteric nerve, a branch of the anterior portion of the mandibular division (V3) of the trigeminal (TG) nerve (Fig. 13*A*,*B*). Anatomical studies have revealed myofascial trigger points, which represent tender muscle areas that can elicit pain whenever stimulated during normal or pathologic condition. These myofascial trigger points coincide with neuromuscular junctions at the innervation zone between MM heads (Procopio Pinheiro et al., 2020). Previous observations of the head and neck area showed the presence of nociceptive unmyelinated C- and myelinated Aδ fibers (Strassman et al., 1996; Strassman and Raymond, 1999), as well as low-threshold mechanoreceptors (LTMRs) consisting of myelinated Aα and Aβ fibers (Lobbezoo et al., 2002). Similarly, it has been shown that MM has C-fibers, two types of A-δ high-threshold mechanoreceptors (Aδ-HTMR) as well as Aβ-LTMR fibers (Nishimori et al., 1986; Cruccu et al., 1989; Connor et al., 2005; Wong et al., 2014; Sato et al., 2018). MM is predominantly innervated by Aδ-HTMR, with cell bodies observed to be 27% small, 49% medium, and 24% large-sized neurons (Sato et al., 2018). However, the precise composition of MM innervating TG ganglion neuronal groups or their properties is unknown.

The understanding of molecular mechanisms governing masticatory myofascial pain is hindered by the limited information on the neuronal circuits undergoing plasticity during TMD and after surgical procedures involving MM and/or masseteric nerve. The aim of the present study has been to identify and comprehensively characterize TG sensory neuronal groups innervating MM (MM TG neurons), and to examine the innervation patterns of MM by afferent sensory fibers. To do so, back-tracing of sensory neurons from MM, patch-clamp electrophysiology, Ca imaging, anatomic studies, sensory neuronal reporter mice and tissues from a nonhuman primate species (common marmosets) have been used (Herrity et al., 2014; Patil et al., 2018).

## Materials and Methods

### Animals

All animal experiments conformed to American Pain Society (APS)’s Guiding Principles in the Care and Use of Vertebrate Animals in Research and Training. We also followed guidelines issued by the National Institutes of Health (NIH) and the Society for Neuroscience (SfN) to minimize the numbers of animals used and their suffering. Protocols used in this study (20190114AR for mice and 20200021AR for nonhuman primates) are approved by the Institution Animal Care and Use Committee (IACUC).

Experiments were performed on 10- to 18-week-old male mice. B6.Cg-Gt(ROSA)26Sortm9(CAG-tdTomato)Hze/J (Ai9; tdTomato; stock 007909), B6.Cg-Gt(ROSA)26Sortm32(CAG-COP4* H134R/EYFP)Hze/J (Ai32; stock 024109), B6;129P2-Pvalbtm1(cre)Arbr/J (PV-cre; stock 008069) and Mrgprd^tm1.1(cre/ERT2)Wql^/J (MrgprD-cre-ER; stock 031286) mouse lines were obtained from The Jackson Laboratory. 5HT3a-GFP (also known as Htr3a-EGFP) and TRPV1-GFP transgenic mouse lines were purchased from the GENSAT program (MMRRC services; UNC, NC and UC Davis, CA, respectively). The CGRP^cre/+-ER^ mouse line was kindly provided by Pao-Tien Chuang (University of California San Francisco, San Francisco, CA). Nav1.8^cre/+^ mouse line was kindly provided by John Wood (University College London, London, United Kingdom). The trkC^cre/+-ER^ mouse line was generated in David Ginty’s laboratory (Harvard Medical School, Boston, MA) and kindly provided by Yu Shin Kim (The University of Texas Health Science Center at San Antonio). In inducible *cre*-carrying mouse lines, *cre*-recombinase was induced in six- to eight-week-old mice by three consecutives (every second day) intraperitoneal injections of 100 mg/kg tamoxifen (dissolved in corn oil). *Cre*-recombination occurs within two to three weeks after tamoxifen injection.

Two aged (11- and 15-year-old) male common marmosets (Callithrix jacchus) were used for collection of MM. IACUC and veterinary oversight regularly monitored marmoset housing and animal conditions to ensure all guidelines for the health and safety of the animals were met and research was conducted in compliance with the United States Public Health Service’s Policy on Humane Care and Use of Laboratory Animals and the Guide for the Care and Use of Laboratory Animals and adhered to the American Society of Primatologists (ASP) principles for the ethical treatment of non-human primates. Marmosets were euthanized for humane reasons following veterinary consult and tissue samples were collected immediately following determination of death.

### Primary TG neuronal culture

To visualize MM TG neurons, wild-type or reporter mice expressing GFP, YFP (Ai32), or tdTomato (Ai9) gene were injected into the right and left MM closer to tendon with 10 μl of WGA-488 or WGA-555 (50 μg in 0.5% DMSO) back-tracer. Neuronal culture for electrophysiology recordings was always generated from one mouse. TG was dissected 24–36 h post-WGA injections, and sensory neurons were cultured as previously described (Belugin et al., 2013). Cells were maintained in DMEM with 2% fetal bovine serum, 2 mm L-glutamine, 100 U/ml penicillin, and 100 μg/ml streptomycin. No growth factor was added to the media. The experiments were performed within 24 h after TG neuron plating. This culture conditions minimize changes in sensory neurons (Patil et al., 2018).

### Electrophysiology: recording

**Figure 1.**
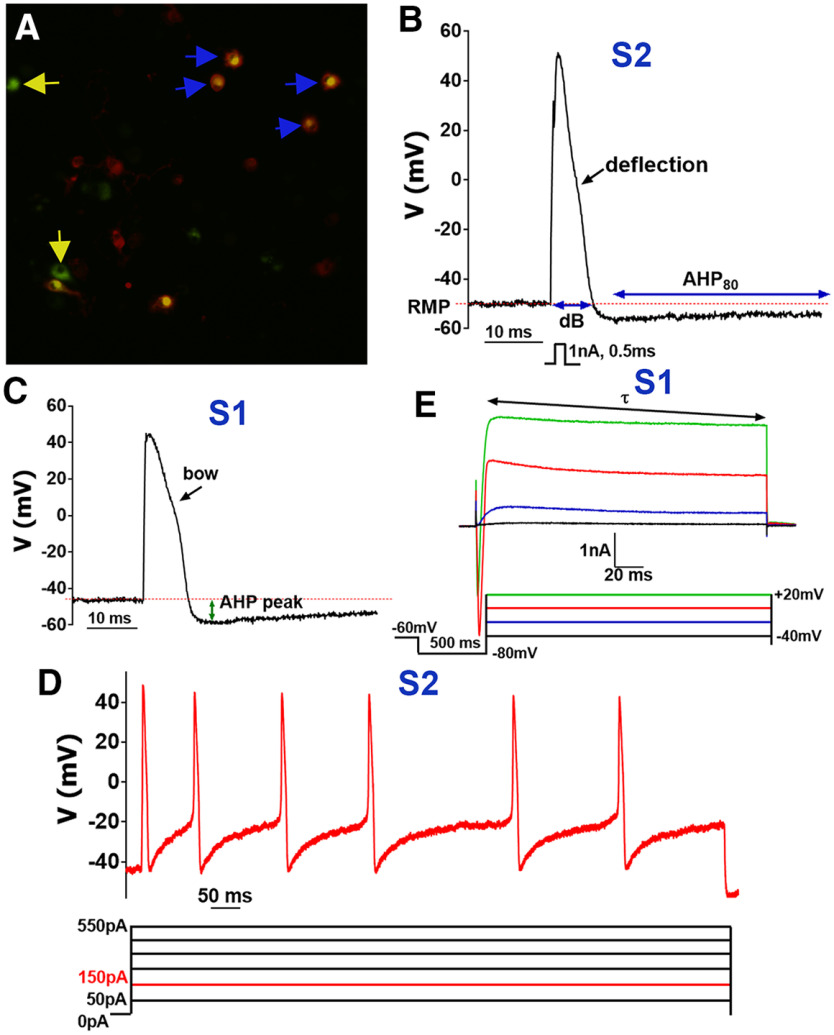
Recordings from non-peptidergic IB4^+^ MM TG neurons. ***A***, WGA-488^+^/IB4-555^+^ (marked with blue arrows), but not WGA-488^+^/IB4^–^ (marked with yellow arrows) were selected for recording non-peptidergic TG neurons innervating MM. ***B***, Stimulus waveform (1 nA, 0.5 ms) indicated below trace generated a single AP in a WGA^+^/IB4^+^ TG neuron belonging to the S2 group (Table 1). AP width is duration at base, dB. AHP80 is the time required for the AHP (measured in mV) to decay by 80% to a RMP level. Characteristic AP deflection is indicated by black arrow. ***C***, AP from a WGA^+^/IB4^+^ TG neuron belonging to the group S1 (Table 1). Distinctive AP feature, bow, is indicated by black arrow. Distance from RMP to a lowest point of AP, AHP peak, is measured as indicated with the green arrow. ***D***, Current-evoked AP train from a WGA^+^/IB4^+^ TG neuron belonging to the S2 group. Current waveforms are below trace and are applied by steps from 50 to 550 pA with 100-pA increment. Depictured AP train is evoked by a 150-pA step lasting 1 s. ***E***, Currents were generated from a WGA^+^/IB4^+^ TG neurons belonging to the S1 group by the indicated waveforms found below traces. The decay constant τ was derived from standard single exponential fits between points indicated by arrows for the outward portion of the final current trace (+20 mV).

Before patch clamp recording, cultured TG cells from WGA-488-injected wild-type mice were stained for 0.5–4 h with IB-4 Alexa Fluor 594 (1:1000; Thermo-Fisher Scientific). WGA^+^/IB-4^+^ TG neurons were selected for recording (Fig. 1*A*). Along with WGA^+^/IB-4^+^ TG neurons from wild-type mice, WGA^+^/CGRP-cre-ER^+^, WGA^+^/TRPV1-GFP^+^, WGA^+^/5HT3a-GFP^+^, WGA^+^/PV-cre^+^, WGA^+^/trkC-cre-ER^+^, WGA^+^/Nav1.8-cre^+^, and WGA^+^/Nav1.8-cre^–^ MM TG neurons were selected for recordings. Overall, we have chosen reporters that are not affected by early developmental changes (Patil et al., 2018).

**Table 1:** Properties of MM TG neuronal groups

Gr^1^	Marker	*N*	Size; μm^2^	RMP; mV	dB; ms	Shape^3^	AHP_80_; ms	AHP peak^4^	AP Thr.; pA	AP train; Hz	ATP	5HT; pA	Tau; ms	Shape Curr^5^
S1	IB4 Nav1.8	16	20.5 ± 0.8	−40.4 ± 2.1	11.9 ± 1.1	Bow	35.2 ± 4.9	−14.5 ± 1.2	405 ± 177	10 ± 2.2	0	0	19.2 ± 6.5	Smooth
S2	IB4 Nav1.8	52	19.9 ± 0.4	−41.3 ± 1.0	11.5 ± 0.6	Deflection	67.9 ± 4.0	−19.0 ± 0.8	156.6 ± 20.3	8.3 ± 0.8	0	0	26.3 ± 4.2	Smooth^6^^,^^7^
S3	trpV1 CGRP Nav1.8	47	21.4 ± 0.4	−44.7 ± 1.0	8.7 ± 0.3	Hump	76.3 ± 17.2	−13.7 ± 0.9	109.1 ± 23.4	11.8 ± 2.2	0	0	20.6 ± 2.6	Smooth^7^
S4	trpV1 CGRP Nav1.8	24	21.4 ± 0.9	−43.0 ± 1.1	8.5 ± 0.5	Deflection	63.5 ± 20.5	−10.3 ± 1.4	64.3 ± 14.3	6.3 ± 1.3	0	0	16.9 ± 2.1	Smooth^6^
S5	CGRP 5HT3a trkC	30	21.5 ± 1.0	−44.7 ± 1.5	7.7 ± 0.5	Straight	65.2 ± 16.7	−13.7 ± 1.3	50.0 ± 0	22 ± 3.8	0	195 ± 18	24 ± 4.6	Box like^6^
M1	CGRP 5HT3a	34	29.5 ± 0.9	−52.1 ± 1.1	6.3 ± 0.2	Deflection	92.4 ± 11.7	−13.2 ± 1.2	316 ± 107	10 ± 3.7	0	818 ± 100	3.5 ± 0.6	Box like^6^
M2^9^	CGRP 5HT3a^8^ trkC PV	66	30.7 ± 0.5	−51.6 ± 1.6	4.8 ± 0.2	Straight	73.6 ± 9.1	−13.3 ± 0.5	580 ± 128	13.1 ± 3.3	0	774 ± 88	22.0 ± 2.3	Spike
M3	trkC PV	56	33.5 ± 0.6	−55.2 ± 0.6	2.8 ± 0.1	Straight	86.3 ± 12.0	−10.3 ± 0.6	1280 ± 152	7 ± 2.3	0	0^11^	22.3 ± 1.9	Spike^10^
M4	trkC PV	40	30.5 ± 0.8	−54.0 ± 0.7	1.9 ± 0.06	Straight	*6.9 ± 0.6*	−10.7 ± 0.8	0	0	0	0	16.0 ± 2.7	Box like

1 Green font is non-peptiderigic; red font is peptidergic, and blue font is mechanoreceptors.

2 Size in μm is calculated from pF (see Materials and Methods).

**Figure 2.**
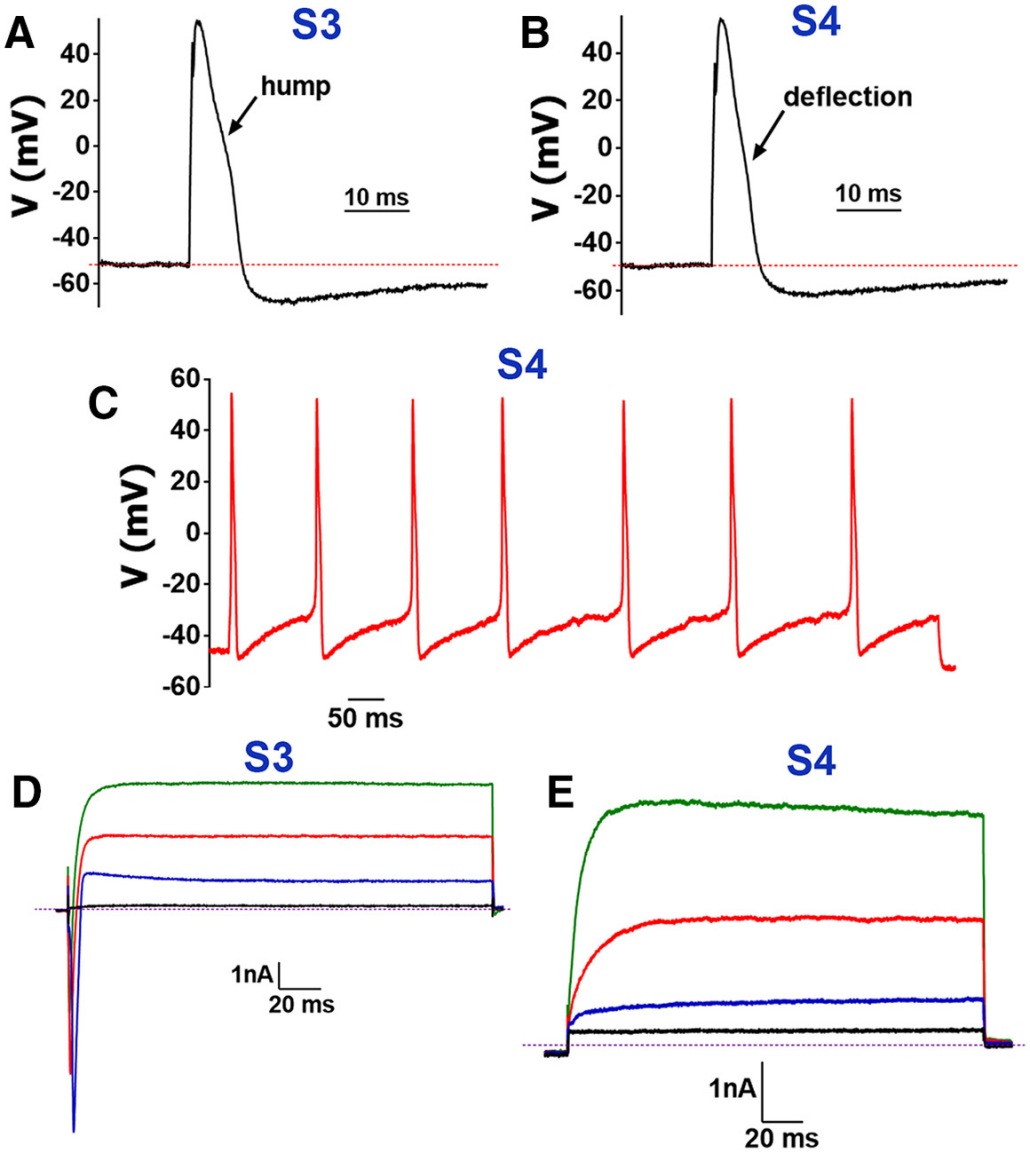
Recordings from TRPV1^+^ MM TG neurons. ***A***, In MM TG neurons belonging to the group S3, a single AP in WGA^+^/TRPV1^+^ neuron is generated by current pulse (protocol-1), which has characteristic hump on down stroke phase of AP. ***B***, In MM TG neurons belonging to the group S4, a single AP in WGA^+^/TRPV1^+^ neuron has distinctive deflection on down stroke phase of AP. ***C***, Current-evoked AP train from a WGA^+^/TRPV1^+^ TG neuron belonging to the group S4. Depictured AP train is evoked by a 50-pA step lasting 1 s. ***D***, In WGA^+^/TRPV1^+^ TG neurons belonging to the group S3, currents were generated by waveforms from Figure 1*E*. ***E***, In WGA^+^/TRPV1^+^ TG neurons belonging to the group S4, currents generated by waveforms from Figure 1*E* do not have inward component.

3 Characteristic feature on AP downstroke: bow, hump, deflection, and straight (Figs. 1*B,C*, 2*A*).

4 AHP_80_ peak is a lowest point of AP, and it is measured in negative mV.

**Figure 3.**
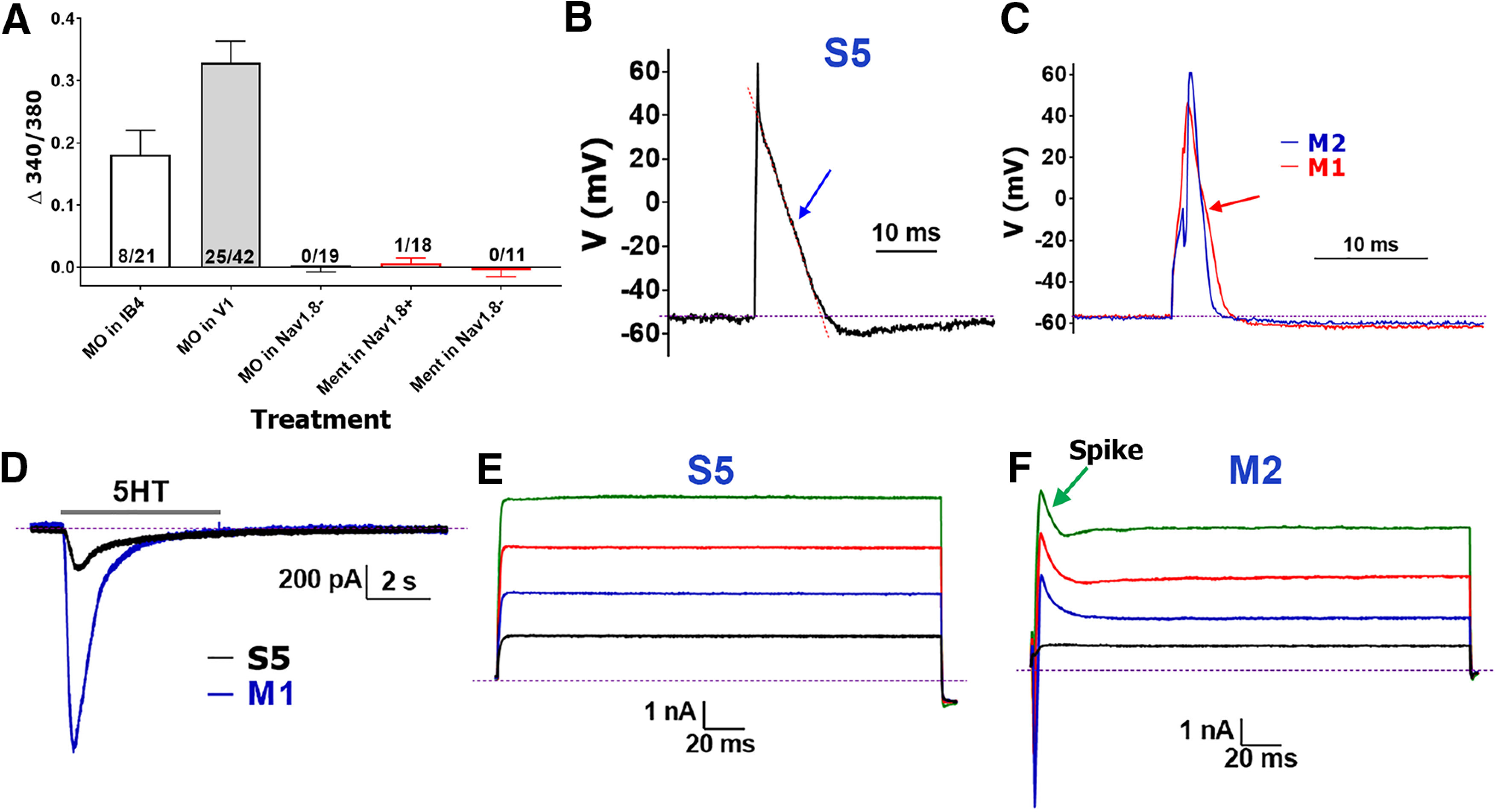
Recordings from CGRP^+^ MM TG neurons. ***A***, Responsiveness of IB-4^+^, Nav1.8^+^, and Nav1.8^–^ MM TG neurons to MO (25 μm) and Ment (100 μm) measured with Ca^2+^ imaging. Each bar shows information on numbers of tested and responsive neurons. ***B***, Representative AP from a MM TG CGRP^+^ neuron belonging to the S5 group. AP straight in S5 neurons is indicated with a blue arrow. Red dashed line demonstrates the AP shape. ***C***, Comparison of AP shapes generated in MM TG CGRP^+^ neurons belonging to the M1 (red) and M2 (blue) groups. AP deflection in M1 neurons is indicated with a red arrow. M2 neuron’s AP does not display any deflection during the falling phase of AP. ***D***, 5HT (30 μm)-induced current in MM TG CGRP^+^ S5 and M1 group neurons. The drug was applied for 5 s, and duration of application is indicated by a horizontal gray bar. ***E***, Typical current (I) produced from MM TG CGRP^+^ neurons belonging to the S5 or M1 group. ***F***, Typical I produced from MM TG CGRP^+^ M2 group neurons. Characteristic spike is indicated by green arrow. Names of neuronal groups are specified above traces. The magnitude (vertical) and time (horizontal) scale bars are presented for the ***B–F*** panels.

**Figure 4.**
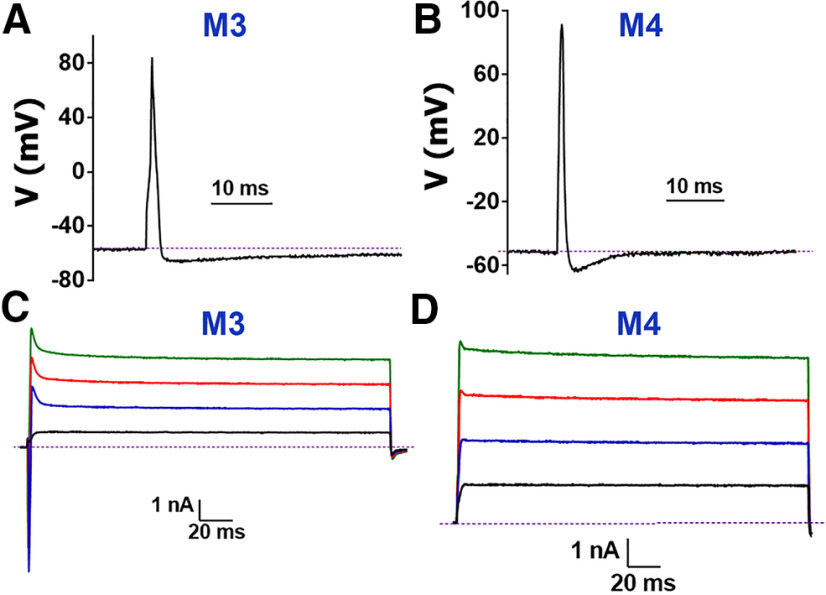
Recordings from Nav1.8^–^ and trkC^+^ MM TG neurons. ***A***, Representative AP from a MM TG trkC^+^ neuron belonging to the M3 group. ***B***, Representative AP from a MM TG trkC^+^ neuron belonging to the M4 group. ***C***, Typical I produced from the M3 group neurons recorded either from a MM TG trkC^+^ or Nav1.8^–^ neuron. Characteristic spike is indicated by green arrow. ***D***, Typical I produced from the M3 group neurons recorded from a MM TG trkC^+^ or Nav1.8^–^ neuron. Names of neuronal groups are specified above traces on the ***A–D*** panels. The magnitude (vertical) and time (horizontal) scale bars are presented for the ***A–D*** panels.

5 Characteristic feature of the last (generated by stepping to +20 mV) outward current: “smooth,” “box like,” and “spike” (Figs. 1*E*, 2*D,E*, 3*E,F*, 4*C,D*).

6 Currents do not have inward component.

7 Neurons are responsive to MO.

8 5HT3a is encoded by Htr3a gene.

9 Contain low level Nav1.8.

10 Spike is sharper in M3 than M2.

11 Some (≈15%) M3 neurons respond to 5HT (30 μm).

Recordings were made in patch clamp whole-cell voltage [holding potential (V_h_) of –60 mV] or current clamp configurations at room temperature. Data were acquired using an Axopatch 200B amplifier and analyzed with pCLAMP10.6 software (Molecular Devices). Recording data were filtered at 0.5–5 kHz and sampled at 2–20 kHz depending on current kinetics. Borosilicate pipettes (Sutter) were polished to resistances of <5 MΩ. If required, access resistance (R_s_) was compensated (40–80%) to the value of <6–8 MΩ. Data were rejected when R_s_ changed >20% during recording, leak currents were >100 pA, or input resistance was <300 MΩ. Liquid junction potential (LJP) was not corrected using the V_meter_ = V_cell_ + LJP equation, since large anions were not included in extracellular or pipette recording solutions. Recordings were made 3–4 min after establishing whole-cell configuration to allow for equilibrium. Currents were considered positive when their amplitudes were fivefold bigger than displayed noise (in root mean square).

Standard external solution (SES) contained the following: 140 mm NaCl, 5 mm KCl, 2 mm CaCl_2_, 1 mm MgCl_2_, 10 mm D-glucose, and 10 mm HEPES, pH 7.4. The standard pipette solution (SIS) contained the following: 140 mm KCl, 1 mm MgCl_2_, 1 mm CaCl_2_, 10 mm EGTA, 10 mm D-glucose, and 10 mm HEPES, pH 7.3, 2.5 ATP and 0.2 GTP. Drugs were applied by a fast, pressure-driven and computer controlled four-channel system (ValveLink8; AutoMate Scientific) with quartz application pipettes. Recordings were independently performed by two investigators. Data were accumulated from five to eight independent MM TG neuronal cultures for each mouse line. Six to 15 neurons were recorded from each MM-TG neuronal culture.

### Electrophysiology: recording protocols and data analysis

On the selected for recording MM TG neurons, we used a sequence of protocols after fast and slow capacitance compensations: (1) single action potential (AP) in current-clamp configuration was generated with 1 nA (2 nA for >40-pF cells) 0.5-ms current pulse (Fig. 1*B*,*C*; Petruska et al., 2000; Patil et al., 2018); (2) AP train was induced by applying step currents of 50–550 pA with 100-pA increment for 1 s (Fig. 1*D*) or 200–2000 pA with 300-pA increment for medium-to-large neurons; (3) after current clamp recordings the electronics were switched to voltage-clamp configuration (V_h_ = –60 mV) and ATP (30 μm) current was recorded by applying drug for 5 s; (4) after ATP-gated current, 5HT (30 μm)-gated current was recorded by 5-s-long drug application; (5) the next protocol in a voltage-clamp configuration was a step down from V_h_ to –80 mV kept for 500 ms, and then 200-ms depolarizing command steps (20 mV) were applied from –40 mV to a final potential of +20 mV (Fig. 1*E*; Petruska et al., 2000). Time gap between these successive one to five protocols was 1–2 min. In some sets of experiments, mustard oil (MO; 25 μm) and menthol (Ment; 100 μm) responses in MM TG neurons of reporter mice were evaluated using Ca^2+^ imaging system as previously described (Salas et al., 2009; Patil et al., 2018).

Data on sensory neuronal marker expression in MM TG neurons, capacitance (in pF) and resting membrane potential (RMP; V_m_ in mV) values were collected prior recording and after application of protocol-1 (Fig. 1*A*,*B*). Cells were considered as a spherical, and therefore, diameter (d in μm) of cells was calculated from capacitance (C_m_ in pF) values using the following:

d=5*√(Cm/4π).

AP duration at the base (dB; time from V_m_ starting point to V_m_ levels at falling phase of AP), after-hyperpolarization (AHP) peak as a distance from RMP to a lowest point of AP and 80% recovery time of AHP to baseline (AHP_80_) were measured from data generated by protocol-1 (Fig. 1*B*,*C*; Table 1). Besides these AP parameters, we noted characteristic features of AP shapes, such as “hump,” “bow,” “deflection,” and “straight,” on the falling phase of AP (Patil et al., 2018; Figs. 1*B*,*C*, 2*A*,*B*, 3*B*,*C*; Table 1). Analysis of protocol-2 gave AP activation threshold (in pA) and maximum AP frequency at the given current input (Fig. 1*D*; Table 1). Protocol-3 and protocol-4 revealed algesic responses to ATP and 5HT as well as I_ATP_ and I_5HT_ characteristics (Fig. 3*D*; Table 1). From protocol-5, the trace evoked by +20 mV was fit with a standard (i.e., single or double) exponential function using the following:

A1 exp[−(t−k)/]+ C.

Fitting and decay *tau* (τ; ms) calculation was performed using pCLAMP10.6 software (Fig. 1*E*). Shape of current (I), including presence or absence of “spike-like” feature at steps to 0 and +20 mV, was an important clustering variable (Figs. 3*F*, 4*C*). Clustering is based on at least two unique features for a specified cluster (Table 2). An approach for generation of clustering parameters described in detail in Results and in the previous publication (Patil et al., 2018).

**Table 2:** Clustering parameters for MM TG neuronal groups

Gr	Key clustering parameters	Putative function
S1	IB4^+^/CGRP^–^; non-responsive to MO and CAP	Non-peptidergic; C-nociceptor
S2	IB4^+^/CGRP^–^; responsive to MO but not to CAP	Non-peptidergic; C-nociceptor
S3	TRPV1^+^/CGRP^+^; AP hump	Peptidergic; C-nociceptor
S4	TRPV1^+^/CGRP^+^; AP deflection; no inward current	Peptidergic; C-nociceptor
S5	CGRP^+^/5HT3a^+^/Nav1.8^–^; AP straight; box-like current; small response to 5HT	?
M1	CGRP^+^/Nav1.8^+^; AP deflection; box-like current; big response to 5HT	Aδ-nociceptors
M2	CGRP^+^/trkC^+^/Nav1.8^–^; AP straight; spike on current; big response to 5HT	?
M3	trkC^+^/CGRP^–^/Nav1.8^–^; AP fast and straight; sharp spike on current; non-response to 5HT	Aβ-mechanoreceptor
M4	trkC^+^/CGRP^–^/Nav1.8^–^; fastest AP; unique AHP; box-like current; non-response to 5HT	Aβ-mechanoreceptor

### Immunohistochemistry (IHC)

Wild-type male C57/Bl mice were injected into the right and left MM close to the tendon with 10 μl of WGA-488 (50 μg in 0.5% DMSO) back-tracer. TG was dissected at 2 d post-WGA injection from 4% paraformaldehyde-perfused mice. MM was dissected from WGA un-injected wild-type mice. TG and MM were collected from two aged marmosets (11 and 15 years of age) that were not injected with WGA. TG and MM from both species were additionally fixed with 4% paraformaldehyde for 15 min, cryo-protected overnight with 30% sucrose in phosphate buffer, embedded in Neg 50 (Richard Allan Scientific), and 25-μm transverse cryo-sections were generated from both mouse and marmoset TG and MM as previously described (Belugin et al., 2013). IHC was conducted as described (Belugin et al., 2013). IHC was simultaneously performed on 6–12 sections generated from three or four mice; or three to four sections generated from the marmoset tissue. The following previously characterized primary antibodies were used for mouse sections: anti-neurofilament heavy chain (NFH) chicken polyclonal antibodies (BioLegend, catalog #PCK-592P, 1:400; Zappulo et al., 2017); anti-pgp9.5 (Millipore-Sigma, catalog #AB1761-I, 1:1000; Roy et al., 2020); anti-TRPV1 guinea pig polyclonal (Neuromics, catalog #GP14100, 1:700; Patil et al., 2014); anti-CGRP rabbit polyclonal (Sigma, C8198, 1:300; Ruparel et al., 2008; Neeb et al., 2011; Lorenzo et al., 2014); anti-tyrosine hydroxylase (TH) rabbit polyclonal (Pel-Freez, P40101, 1:400; Dunkley et al., 2004; Ma et al., 2004); anti-mrgD rabbit polyclonal (Alamone Lab, AMR-061, 1:200; Chang et al., 2016; Patil et al., 2018); anti-trkC goat polyclonal (R&D Systems, AF1404, 1:200; Arcourt et al., 2017; Patil et al., 2018); anti-trkB goat polyclonal (R&D Systems, AF1494, 1:200; Ninkina et al., 1997; Arcourt et al., 2017); rabbit anti-parvalbumin (Swant, PV25, 1:500; Schurmans et al., 1997; Schwaller et al., 1999; Arcourt et al., 2017); and rabbit anti-Calbindin D28k (Swant, CB-38a, 1:500; Schurmans et al., 1997; Arcourt et al., 2017). Antibodies for MrgprD and PV produced weaker signals compare to other antibodies and GFP/YFP/tdTomato signals, hence their intensities were increased, and backgrounds were reduced. Anti-NFH and anti-pgp9.5, but not anti-CGRP produced clear IHC signals in marmoset sections (Resnikoff et al., 2019). Sections were incubated with species appropriate Alexa Fluor secondary antibodies (1:200; Invitrogen).

Images were acquired using a Keyence BZ-X810 All-in-One Fluorescent Microscope (Keyence) or a Nikon Eclipse 90i microscope (Nikon Instruments) equipped with a C1si laser scanning confocal imaging system. Images were processed with NIS-elements (Nikon Instruments) and Adobe Photoshop CC 2019 software. Gain setting was constant during acquisition, and it was established on no primary control slides. Control IHC was performed on tissue sections processed as described but either lacking primary antibodies or lacking primary and secondary antibodies. Cell counts from IHC images acquired as Z-stuck were performed using ImageJ software. Cells positive for WGA and each marker as well as the combinations of markers were counted. We used three or four independent mice (*n* = 3–4) to generate sections and counted three to five sections per mouse. Mean values from these three to five sections represented *n* of 1.

### Statistical analysis

GraphPad Prism 8.0 (GraphPad) was used for statistical analyses. Data in the figures are mean ± SEM, with *n* referring to the number of mice used for IHC and the numbers of analyzed recorded cells. Differences between IHC and electrophysiologically characterized groups were assessed by unpaired *t* test or regular one-way ANOVA with Tukey’s *post hoc* tests, each column was compared with all other columns. A difference is accepted as statistically significant when *p* < 0.05. Interaction *F* ratios and the associated *p* values are reported.

## Results

Sensory neurons have previously been identified and classified using multiple methods including, back-tracing from the target tissue (da Silva Serra et al., 2016), sensory neuron reporter mice, patch-clamp recording and classification according to AP properties, sensitivity to algesic agents and appearances of a variety of voltage-gated currents (Xu et al., 2010; Li et al., 2011; Patil et al., 2018) and IHC (Patil et al., 2018). Described in Materials and Methods, back-tracing from MM will label TG neurons innervating MM fibers, tendon, muscle-tendon junction, and massteric fascia. This approach could have two drawbacks. First, along with these MM structures, diffusion of WGA injected into MM led to labeling of adjusted tissues such as masseteric nerve fibers and the subcutaneous layer of facial skin (Fig. 13*A*,*B*). Second, WGA produces nonuniform size-dependent labeling of neurons (Robertson, 1990). This nonuniform labeling of TG neurons is unlikely miss entire neuronal group since there is a variation in neuronal sizes within every TG neuronal group. Patch clamp experiments from these WGA-labeled MM TG neurons will yield data on 12 variables: cell size, RMP, AP width (dB), characteristic features of AP shapes, AHP-peak, AHP_80_, responsiveness to 5HT, ATP, capsaicin (CAP), MO, and Ment, τ (*tau*) from fitting of voltage-gated currents (I), and shape of these I, including presence or absence of a spike-like feature on outward portions of these voltage-gated currents (Table 1).

### IB4^+^ MM TG sensory neuronal groups

Cultured TG neurons from wild-type male mice back-traced with WGA-488 from MM were stained with IB-4–555 for 0.5–4 h (Patil et al., 2018). WGA^+^ cells considered IB-4^+^, when strong and clear plasma membrane staining was detected (Fang et al., 2006; Fig. 1*A*). These strongly stained IB-4^+^ neurons have been considered as non-peptidergic (Stucky and Lewin, 1999). Seventy-one WGA^+^/IB-4^+^ MM TG neurons (Fig. 1*A*) were recorded with sequential protocols as described in the Materials and Methods. All but three IB-4^+^ MM TG neurons could be assigned to one of two clusters (S1 and S2; Table 1). S1 and S2 have many similar features such as size, RMP, broad AP (i.e., high dB values; Fig. 1*B*,*C*), AHP_80_, time to AHP peak, non-responsiveness to ATP, 5HT and CAP, capability to produce evoked AP train (Fig. 1*D*) and τ values (Table 1). S2 IB-4^+^ MM TG neurons are distinct from S1 because of the following three findings: only S2 neurons were responsive to MO (25 μm; TRPA1 agonist; Fig. 3*A*); S2 has deflection on AP (Fig. 1*B*), while S1 has bow (Fig. 1*C*); and unlike S1 but like S4, current (I) in S2 neurons had no inward component (compare Figs. 1*E* and 2*E*). S1 and S2 are different from other types of MM TG neurons as we observed staining with IB-4, but no CGRP^+^ labeling (i.e., IB-4^+^/CGRP^–^ neurons). Overall, we observed two subtypes of non-peptidergic small-sized TG neurons labeled after back-tracing with WGA from MM.

### TRPV1^+^ MM TG sensory neuronal groups

Fifty-five TRPV1^+^ MM TG neurons (WGA^+^/TRPV1^+^) were recorded from TRPV1-GFP reporter mice (Patil et al., 2018). Two groups (S3 and S4) were delineated. S3 and S4 had several common electrophysiological properties: size, RMP, broad AP, AHP_80_, time to AHP peak (Fig. 2*A*,*B*), non-responsiveness to ATP and 5HT, capabilities to produce evoked AP train (Fig. 2*C*) and τ values (Table 1). We found S3 TRPV1^+^ MM TG neurons were differentiated from S4 because of the following: only S3 neurons were responsive to MO (Fig. 3*A*); S3 had a hump (more pronounced than deflection) on AP (Fig. 2*A*), while S4 had a deflection (Fig. 2*B*); and unlike S4, current (I) in S3 neurons had a large inward component (compare Fig. 2*D*,*E*). S3 and S4 are dissimilar from other types of TG neurons back-traced from MM in expression of TRPV1 and responsiveness to CAP (Table 2). In summary, there are two subtypes of TRPV1^+^ small-sized TG neurons labeled by WGA injected into MM.

### CGRP^+^ MM TG sensory neuronal groups

A total of 122 CGRP^+^ MM-TG neurons were recorded and analyzed. We selected approximately equal numbers of small-sized (<30 pF) and medium-to-large-sized (>30 pF) neurons for recording. A majority (48 from 62) of small-sized (<30 pF) WGA^+^/CGRP^+^ neurons were CAP responsive and according to their properties, could be classified as either S3 or S4 (Table 1). This indicated that all TRPV1^+^ MM TG neurons were peptidergic small-sized neurons. Fourteen small-sized WGA^+^/CGRP^+^ neurons, clustered as S5, had unique characteristics: straight shape on falling phase of AP (Fig. 3*B*), small 5HT-evoked current (Fig. 3*D*), no response to CAP, MO, and Ment (TRPM8 agonist), and “box”-shaped current without an inward component (Fig. 3*E*). Moreover, S5 neurons readily generated an AP train even after 50-pA current injection (Table 1).

Sixty recorded and analyzed medium-to-large-sized CGRP^+^ MM TG neurons could be divided into two groups, M1 and M2. Unlike all small-to-large-sized (i.e., S1–S5) and other medium-to-large-sized (i.e., M3 and M4) MM TG neurons, M1 and M2 possessed a large 5HT-evoked inward current (Fig. 3*D*; Table 1). We have additionally recorded from 24 WGA^+^/5HT3a^+^ neurons cultured from 5HT3a-GFP reporter mouse TG. All recorded neurons were classified as either S5, M1 or M2, and responded to 5HT (Fig. 3*D*). Unlike small-sized MM TG neurons (S1–S5 groups), large current injections were required to produce AP train, which was detected only in 10–20% of M1 and M2 neurons (Table 1). The I (current) recorded from M1 was like S5 neurons, having the lowest τ values among all MM TG neuronal groups (one-way ANOVA; *F*_(8,261)_ = 5.631; *p* < 0.0001; Fig. 3*E*; Table 1). The most distinct features of M2 compare to M1 neurons was their significantly narrower AP (*t* test; *t* = 5.149 df = 109; *p* < 0.0001; Fig. 3*C*) and substantially different shape of current (I) with spike and large inward component (compare Fig. 3*E*,*F*). These characteristics indicate that M1 and M2 belong to medium-sized peptidergic TG MM neurons.

### Nav1.8^–^, trkC^+^, and PV^+^ MM TG sensory neuronal groups

Eighty-six Nav1.8^–^ MM TG neurons were recorded and analyzed. All 16 small Nav1.8^–^ MM TG neurons (<30 pF) belonged to the S5 neuronal group. Twenty-six Nav1.8^–^ MM TG neurons were classified as M1 (6 neurons) or M2 (20 neurons). The remaining neurons could fit into two clusters – M3 and M4 (Tables 1, 2). Size, RMP, AHP peak, AHP_80_, τ, presence of spike on the I (Figs. 3*F*, 4*C*) and requirement of large current to generate AP train were similar for M3 versus M2 neurons (Table 1). An exception was AP dB, which was significantly narrower for M3 compared with M2 neurons (*t* test; *t* = 9.622 df = 125; *p* < 0.0001; Fig. 4*A*). However, the most critical parameter distinguishing M3 from M2 was their responsiveness to 5HT, which was detected exclusively in M2 neurons (Tables 1, 2). M4 neurons had several unique properties distinguishing them from other MM TG neurons. First, no AP train could be generated even with an injection of 2-nA current (protocol-2). Second, M4 neurons were fastest and had the smallest dB value (one-way ANOVA; *F*_(8,347)_ = 91.21; *p* < 0.0001; Fig. 4*B*; Table 1). Third, AHP_80_ was fastest in M4 neurons (one-way ANOVA; *F*_(8,279)_ = 6.398; *p* < 0.0001; Fig. 4*B*; Table 1). Fourth, the I from M4 neurons had a “box-like” shape but possessed a small spike (Fig. 4*D*). We also recorded algesic currents from medium-to-large sized MM TG neurons. These neurons did not have ATP, MO, and Ment-gated currents (Fig. 3*A*; Table 1).

Next, using reporter mice, we recorded and analyzed 65 trkC^+^, 32 PV^+^, and 35 medium-to-large sized Nav1.8^+^ MM TG neurons. This information revealed that trkC was expressed by S5, M2–M4 neurons; PV was present in M2–M4 groups; and Nav1.8 was mainly detected in S1–S4 groups. Medium-to-large sized Nav1.8^+^ MM TG neurons had weaker YFP signal than Nav1.8^+^ small-sized neurons. A bulk majority of medium-to-large sized Nav1.8^+^ MM TG neurons belonged to the M1 group, but four of 35 were classified into the M2 group. Since M1 and M2 neurons were recorded among Nav1.8^+^ and Nav1.8^–^ neurons, this suggests that they had weak Nav1.8 expression, which was sufficient to drive Ai32 reporter in some, but not for all M1 and M2 neurons.

Figure 13*C* summarizes clustering of TG neurons labeled by back-tracing from MM. These clusters were generated on this basis of electrophysiological properties obtained from recordings on TG neurons isolated from different reporter mouse lines. Unlike previously characterized L3–L5 DRG neurons, which innervate the skin and muscle of legs, TG neurons labeled by back-tracing from MM did not have small-sized TRPV1^+^/CGRP^–^ (somatostatin^+^) neurons (Usoskin et al., 2015; Sharma et al., 2020), TRPM8^+^ (cold thermoceptors) neurons (Sharma et al., 2020) and strongly responding to ATP MrgprA3^+^ neurons (Usoskin et al., 2015; Patil et al., 2018). MM TG neurons also did not have multiple small-sized peptidergic groups (Patil et al., 2018; Zeisel et al., 2018). Expectedly, MM TG lacked neuronal groups associated with innervation of hairs: C-LTMR and Aδ-LTMR, which are Nav1.8^+^/CGRP^–^/trkC^–^ (Usoskin et al., 2015) and have distinct AP (Patil et al., 2018). Finally, MM TG neurons do not have proprioceptors (Sharma et al., 2020), which are located in a brain stem region of the TG system. Overall, MM TG neuronal groups were found to be substantially different from the well-characterized L3–L5 DRG neuronal clusters, and S5 and M2 groups have no analogs among L3–L5 DRG neurons (see Discussion).

### IHC analysis of MM TG sensory neuronal groups

**Figure 5.**
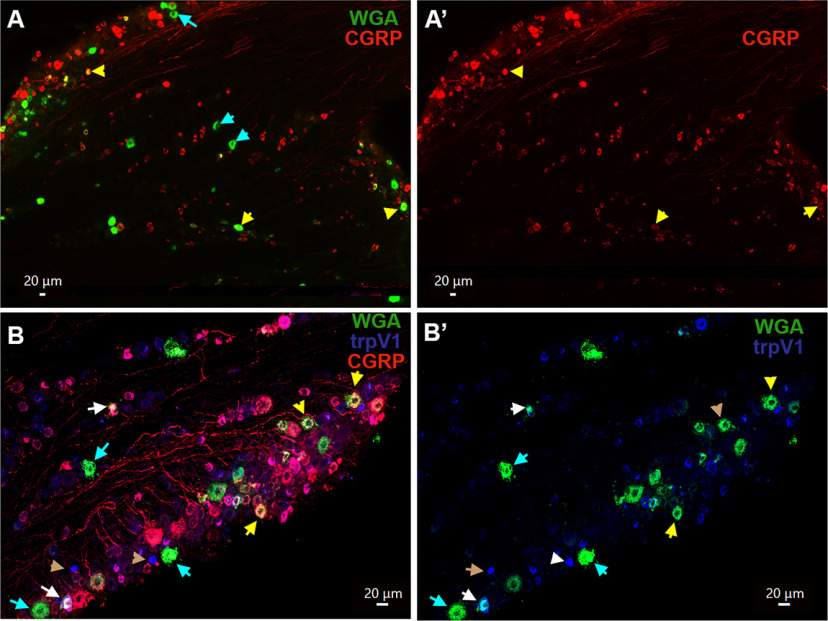
Expression of CGRP and TRPV1 in MM TG neurons. ***A***, ***A’***, Expression of CGRP (red) neurons in a TG section containing MM TG neurons (WGA; green). Objective is 10×. ***B***, ***B’***, Expression of CGRP (red) and trpV1 (blue) neurons in a TG section containing MM TG neurons (WGA; green). Objective is 20×. MM TG neurons lacking marker are marked with cyan arrows on the ***A–C*** panels. MM TG neurons containing both WGA and CGRP or trpV1 are marked with yellow arrows. MM TG neurons containing WGA, but not CGRP or trpV1, are marked with cyan arrows. MM TG neurons containing both CGRP and trpV1 are marked with white arrows on the ***B***, ***B’*** panels. TrpV1 neurons lacking CGRP and WGA are marked with brown arrows on the ***B***, ***B’*** panels. White horizontal bar shows 20-μm scale for each panel.

To further characterize the MM TG sensory neuronal groups and confirm our electrophysiology data, we used IHC to examine expression of sensory neuronal markers in TG neuron sections from naive male mice injected with WGA-488 into MM. Peptidergic MM TG neurons (S3–S5, M1, and M2 groups) labeled with CGRP antibodies composed 51.7 ± 2.1% of WGA^+^ TG neurons (Figs. 5*A*,*A’*, 8*C*). Modest 15.3 ± 2.4% of WGA^+^ TG neurons were TRPV1^+^ (S3 and S4 groups) and all of these neurons were labeled with CGRP (Figs. 5*B*,*B’*, 8*C*, 13*C*). Consistent with our electrophysiology data, TRPV1^+^/CGRP^–^ TG neurons, which have strong TRPV1 labeling (Fig. 5*B*,*B’*, brown arrows), did not contain WGA.

**Figure 6.**
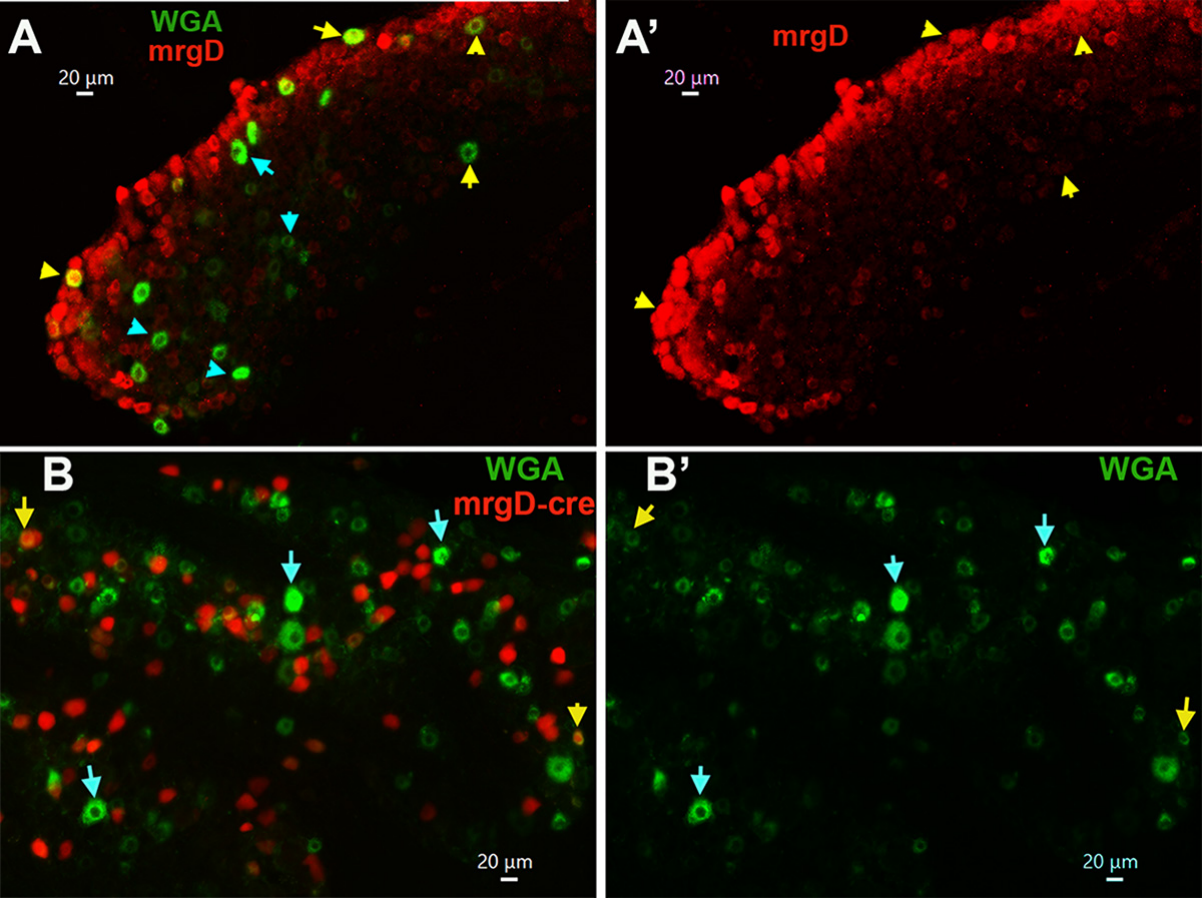
Expression of MrgprD in MM TG neurons. ***A***, ***A’***, Expression of MrgprD (mrgD; red) neurons in a TG section containing MM TG neurons (WGA; green). Objective is 20×. ***B***, ***B’***, Expression of MrgprD-cre/tdTomato (mrgD-cre; red) neurons in a TG section from mice back-traced from MM with WGA-488 (WGA; green). Objective is 20×. MM TG neurons containing both mrgD or mrgD-cre and WGA are marked with yellow arrows. MM TG neurons containing WGA, but not mrgD or mrgD-cre or trpV1, are marked with cyan arrows. White horizontal bar shows 20-μm scale for each panel.

Small-sized non-peptidergic (i.e., IB4^+^/CGRP^–^) DRG neurons express MrgprD as a marker (Reynders et al., 2015; Patil et al., 2018). However, despite 24 ± 2.4% of WGA^+^ cultured TG neurons staining for IB-4 (S1 and S2 groups), only a few (3.1 ± 0.7%, n = 3) MM TG neurons expressed MrgprD (Figs. 6*A*,*A’*, 8*C*). Anti-mrgprD antibodies are weak and generate high background (Fig. 6*A*,*A’*); therefore, we used an alternative approach to confirm these data by back-tracing WGA from MM in MrgprD-cre-ER/tdTomato reporter mice. Analysis of these sections confirmed that the MrgprD-cre^+^ signal was only detected in a few TG MM neurons (2.3 ± 0.6%, n = 3; Fig. 6*B*,*B’*). However, some of MrgprD-cre^+^ signals were registered in weakly labeled WGA^+^ neurons (Fig. 6*B*,*B’*). These data indicate that MrgprD may not be an appropriate marker for non-peptidergic MM TG neurons.

**Figure 7.**
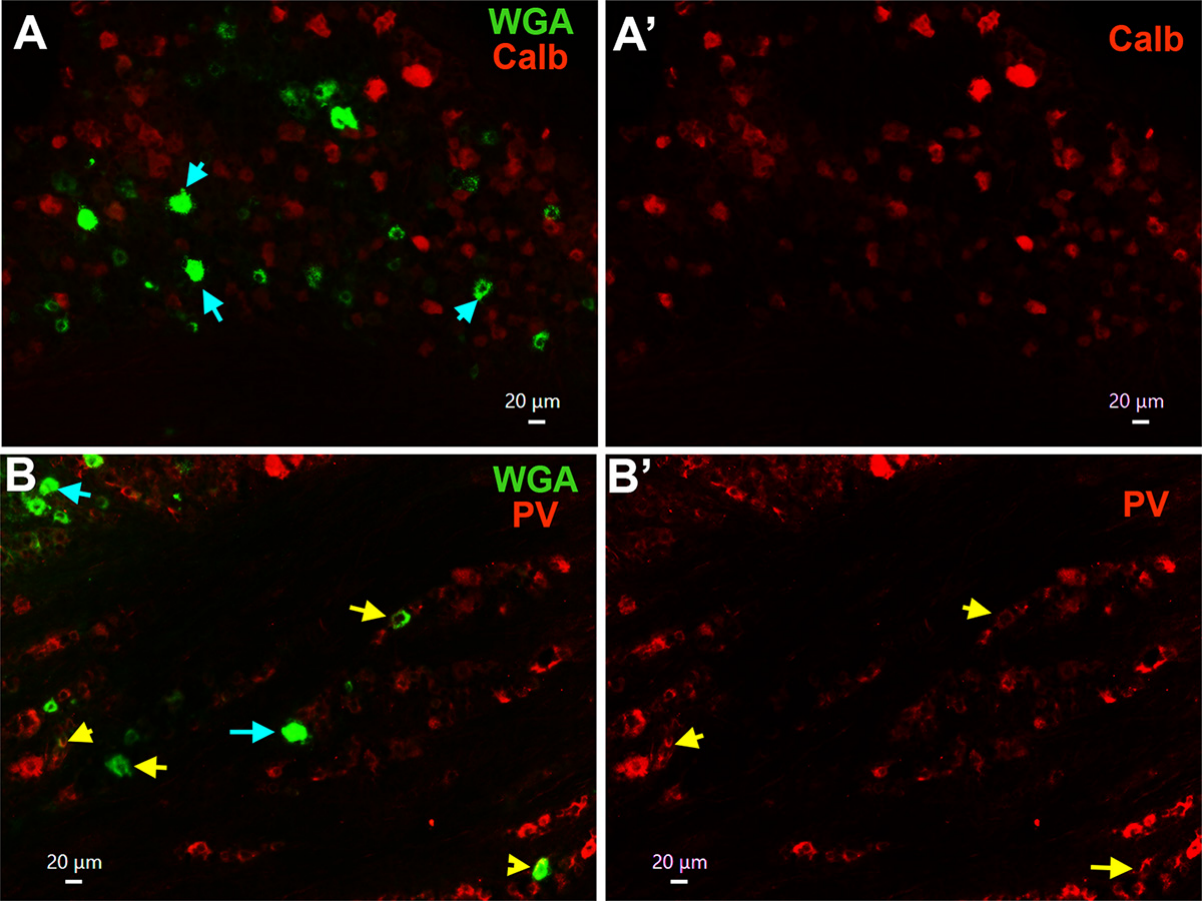
Expression of calbindin-D28 and parvalbumin mechanoreceptor markers in MM TG neurons. ***A***, ***A’***, Expression of calbindin D28 (Calb; red) in a TG section containing MM TG neurons (WGA; green). ***B***, ***B’***, Expression of parvalbumin (PV; red) in a TG section containing MM TG neurons (WGA; green). MM TG neurons lacking Calb or PV are shown with cyan arrows. MM TG neurons containing Calb or PV are shown with yellow arrows. For all panels, objective is 20×. White horizontal bar shows 20-μm scale for each panel.

**Figure 8.**
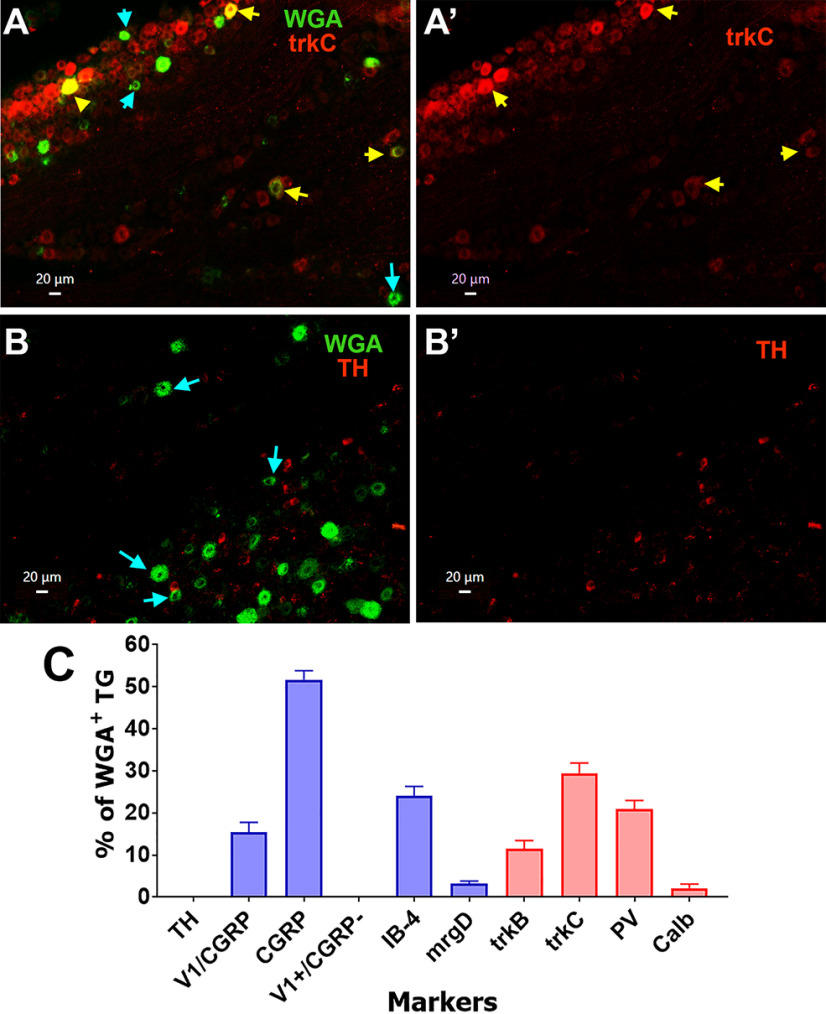
Expression of trkC and TH mechanoreceptor markers in MM TG neurons. ***A***, ***A’***, Expression of trkC (trkC; red) in a TG section containing MM TG neurons (WGA; green). ***B***, ***B’***, Expression of TH (red) in a TG section containing MM TG neurons (WGA; green). MM TG neurons lacking a sensory neuronal marker are shown with cyan arrows on the ***A***, ***B*** panels. MM TG neurons containing a sensory neuronal marker are shown with yellow arrows on the ***A***, ***A’*** panels. For all panels, objective is 20×. White horizontal bar shows 20-μm scale for each panel. ***C***, Percentages of WGA^+^ TG neurons labeled with a variety of indicated sensory neuronal markers. Cell counting is from four animals, three to five sections each.

Single-cell transcriptomic of L3–L5 mouse DRG neurons assigned calbindin D28-positive (Calb^+^) neurons to group NF2 (Usoskin et al., 2015) or Aβ-Field (Sharma et al., 2020). Functional studies on L3–L5 DRG Calb^+^ neurons demonstrated that they are a subset of Aβ-LTMR neurons (Arcourt et al., 2017). TG MM neurons express Calb at low levels (≈2%; Figs. 7*A*,*A’*, 8*C*). Recording from MM TG neurons isolated from PV-cre/tdTomato reporter mice showed that PV could be present in M2–M4 groups. IHC data indicate that PV antibodies had low signal in ≈21% MM TG neurons (Figs. 7*B*,*B’*, 8*C*). Similarly, electrophysiology data on MM TG neurons from trkC/tdTomato mice indicated that trkC could be found in groups S5, M2–M4. TrkC antibodies labeled ≈30% WGA^+^ TG neurons (Fig. 8*A*,*A’*,*C*). This implies that S5 are ∼5% of MM TG neurons (Fig. 13*C*). Our patch-clamp recordings did not show the presence of C-LTMR-like afferents among MM TG neurons. IHC confirmed that TH^+^ (a marker for C-LTMR neurons) was not present among WGA^+^ neurons (Fig. 8*B*,*B’*,*C*). We did not detect any characteristic properties that would point to Aδ-LTMR-like (i.e., trkB^+^ in DRG) afferents among patch-clamp recorded neurons. However, trkB was found to be expressed in 11.5 ± 1.9% of MM TG neurons (Fig. 8*C*). In conclusion, considering the information on expression percentages of WGA^+^/CGRP^+^, WGA^+^/IB-4^+^, and WGA^+^/trkC^+^ as well as frequency of recordings from particular MM TG neuronal groups, we could estimate that 24% of MM TG neurons belong to groups S1 and S2; 15% to S3 and S2; 5% to S5, 24% to M1; 5% to M2; 12% to M3; and 10% to M4 (Fig. 13*C*).

### TG sensory afferent nerve types innervating masseteric muscle fibers and tendons

The masseteric nerve enters the deep portion of MM and is divided into many branches within MM (Fig. 13*A*,*B*). These branches eventually supply the sensory afferent nerves that innervate MM fibers, tendon, muscle-tendon junction, and masseteric fascia. Injection into MM could lead to contamination and/or diffusion of WGA into subcutaneous tissue (Fig. 13*B*). Additionally, such an injection could label sensory afferent fibers in the main trunk of masseteric nerve (Fig. 13*A*,*B*). Using IHC, we examined the distribution of different sensory fiber types within MM, as well as innervation of muscle fibers, tendon, muscle-tendon junctions, and fascia surrounding whole MM.

**Figure 9.**
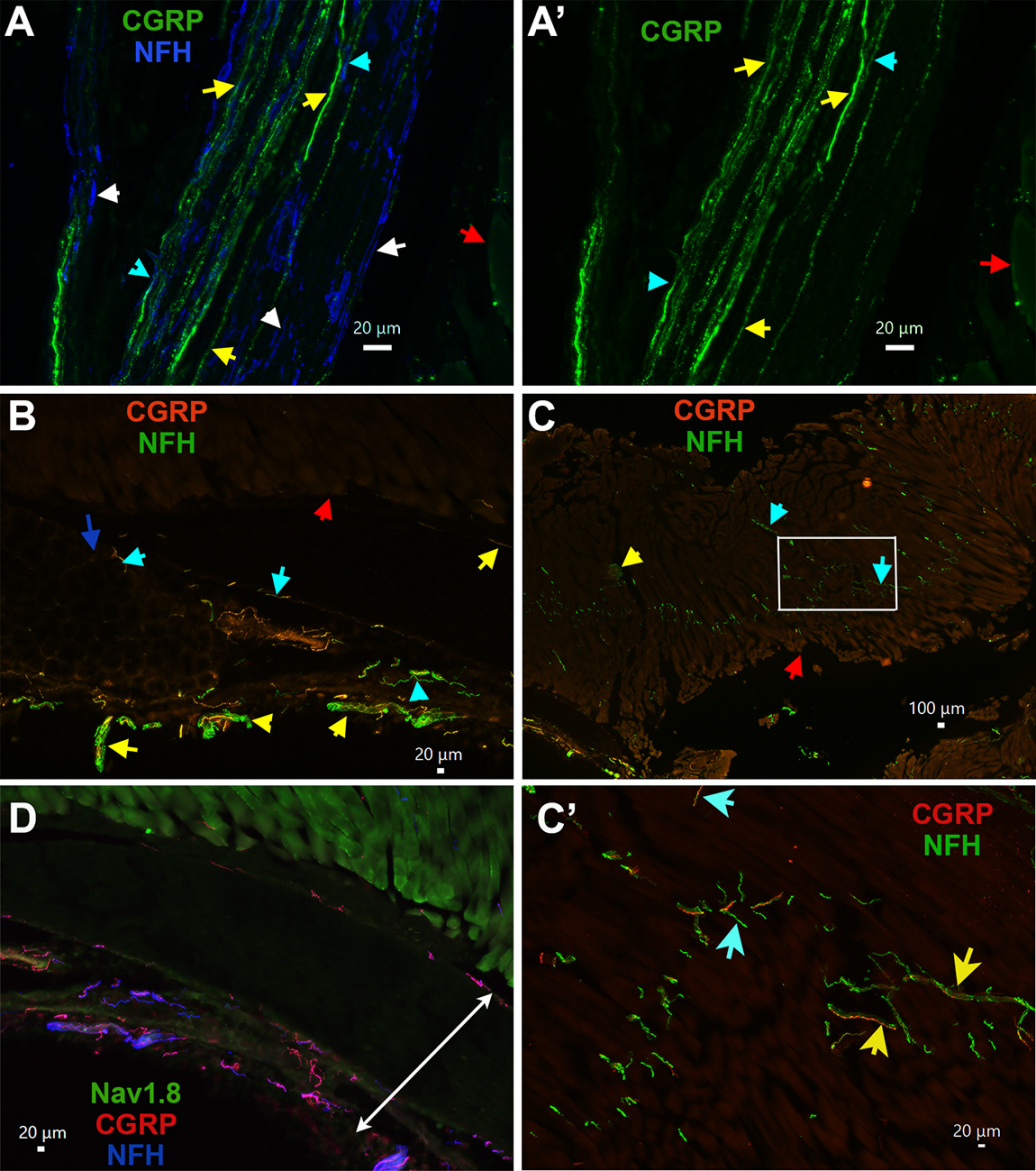
Location of CGRP^+^ and NFH^+^ TG sensory neuronal afferent fibers in MM. ***A***, ***A’***, Expression of CGRP (green) and NFH (blue) fibers in a main trunk of the masseteric nerve located near MM. Objective is 40×. CGRP^+^/NFH^–^ nerves are marked with yellow, CGRP^+^/NFH^+^ with cyan, and CGRP^–^/NFH^+^ with white arrows. Red arrow shows MM fibers. ***B***, Expression of CGRP (red) and NFH (green) nerves in muscle fibers and MM tendon. Objective is 10×. Red arrow shows muscle fibers and blue points on tendons. Nerve bundles are marked with yellow arrows and individual nerves with cyan arrows. ***C***, Expression of CGRP (red) and NFH (green) nerves in MM. Objective is 2×. A red arrow shows muscle fibers. ***C’***, High magnification (objective is 20×) of the image within a white rectangle on the panel ***C***. On the panels ***C***, ***C’***, nerve bundle is marked with a yellow arrow and individual nerves with cyan arrows. ***D***, Expression of Nav1.8-Ai32-YFP (green), CGRP (red), and NFH (blue) nerves in MM fascia area, which is indicated by white double arrowed line. Objective is 10×. White horizontal bar shows 20- or 100-μm scale for each panel.

The main trunk of the masseteric nerve brunches out from the mandibular nerve and enter the MM in the sigmoid fascia area (Kim et al., 2010; Fig. 13*A*,*B*). We found this area of masseteric nerve trunk contained CGRP^+^/NFH^–^ (yellow arrows; S3–S5 groups), CGRP^+^/NFH^+^ (M1 and M2 groups; Fig. 9*A*,*A’*, cyan arrows) and CGRP^–^/NFH^+^ (M3 and M4 groups; Fig. 9*A*,*A’*, white arrows) sensory nerves. The masseteric nerve trunk then descends into MM between the middle and deep parts of muscle fibers and then divides into many nerve branches in the posterosuperior, posteroinferior, anterosuperior, and anteroinferior directions (Luo et al., 1991; Kim et al., 2010; Fig. 13*A*,*B*). We observed that multiple branches of CGRP^+^ and NFH^+^ nerve bundles reached the deep and middle layers of the muscle and junctions of muscle fibers and tendon (Fig. 9*B*). Distribution of CGRP^+^ and NFH^+^ nerve bundles and individual fibers occurred throughout MM. However, they mainly concentrate in tendon and at junctions between superficial, middle, and deep parts of MM (Fig. 9*B*,*C*,*C’*). Additionally, these fibers were found to innervate masseteric fascia, which surrounds the whole MM (Fig. 9*D*).

**Figure 10.**
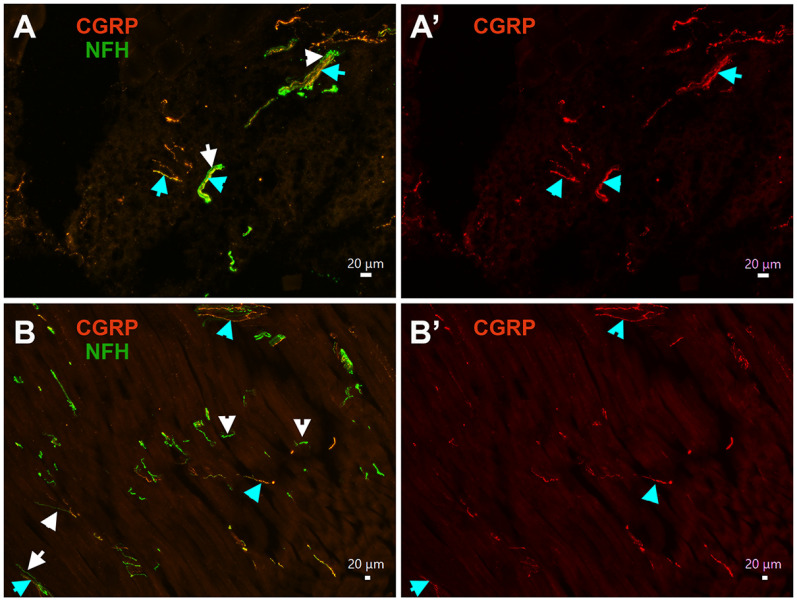
MM TG neuron types innervating mouse MM fibers and tendons. ***A***, ***A’***, Expression of CGRP (red) and NFH (green) nerves in MM tendon. Objective is 20×. CGRP^+^/NFH^+^ nerves are marked with cyan and CGRP^–^/NFH^+^ with white arrows. ***B***, ***B’***, Expression of CGRP (red) and NFH (green) nerves in masseteric muscle fibers. Objective is 10×. CGRP^+^/NFH^+^ nerves are marked with cyan and CGRP^–^/NFH^+^ with white arrows.

Analysis of multiple slides showed that MM tendons almost exclusively contained CGRP^+^/NFH^+^ (M1 and M2 groups; Fig. 10*A*,*A’*, cyan arrows) and CGRP^–^/NFH^+^ nerves (M3 and M4 groups; Fig. 10*A*,*A’*, white arrows). Masseteric muscle fibers were also nearly exclusively innervated by CGRP^+^/NFH^+^ (cyan arrows) or CGRP^–^/NFH^+^ (white arrow) MM TG neurons (Fig. 10*B*,*B’*). This innervation pattern was detected around masseteric fascia as well (Fig. 9*D*). Thus, only 1–3 peptidergic CGRP^+^/NFH^–^ nerves (S3, S4, and maybe S5 groups) were observed in muscle fibers, adjacent tendons of MM and masseteric fascia investigated by 20× objective across 16–24 MM sections generated from three to four mice.

**Figure 11.**
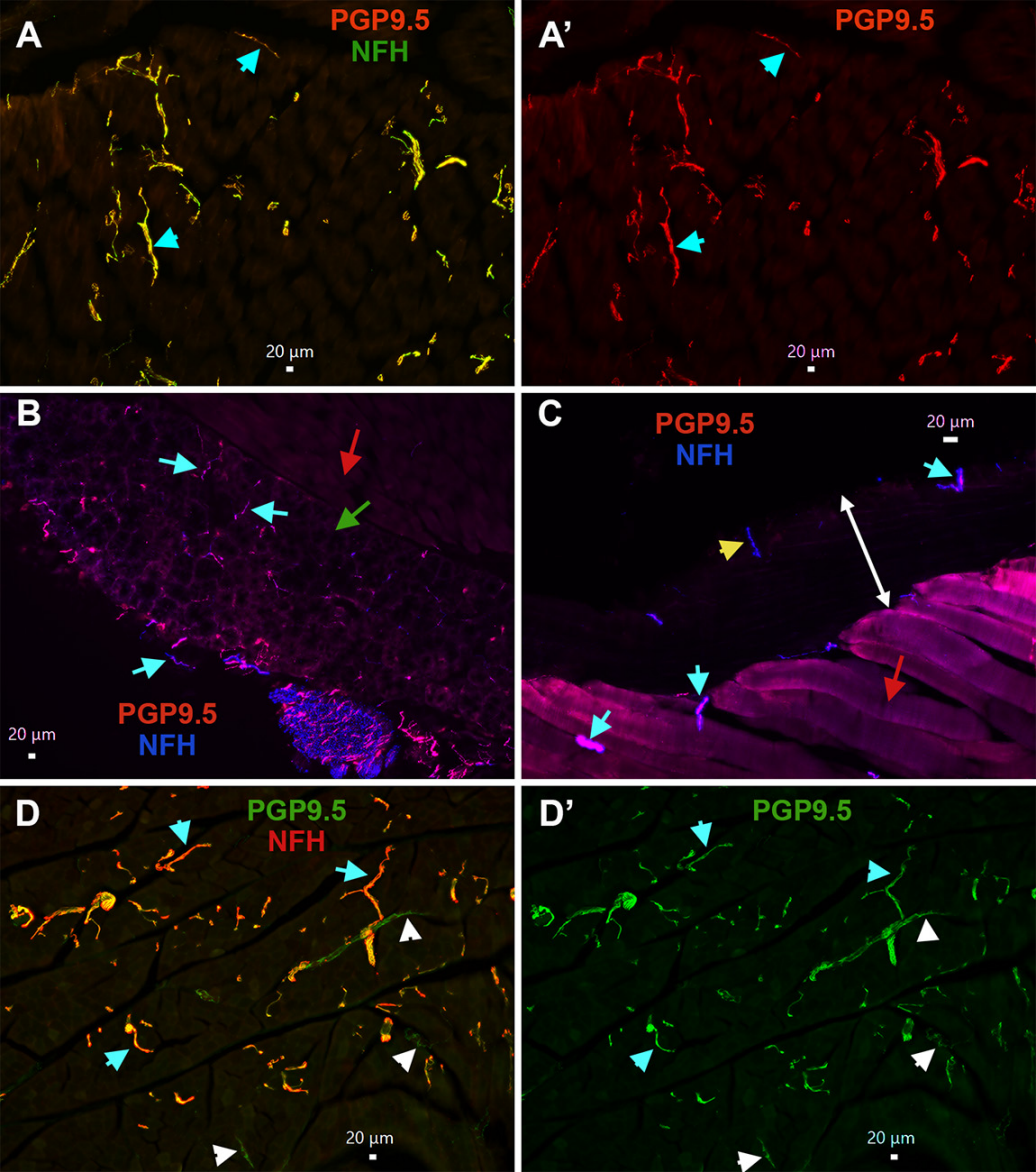
MM TG neuron types innervating MM in mice and common marmosets. ***A***, ***A’***, Expression of pgp9.5 (red) and NFH (green) in male mouse masseteric muscle fibers. Objective is 10×. Pgp9.5^+^/NFH^+^ nerves are shown with cyan arrows. ***B***, Expression of pgp9.5 (red) and NFH (blue) in male mouse MM tendon. Objective is 10×. Muscle fibers are shown by a red arrow, and tendons by a green arrow. Pgp9.5^+^/NFH^+^ nerves are shown with cyan arrows. ***C***, Expression of pgp9.5 (red) and NFH (blue) in male mouse masseteric fascia. Objective is 20×. Muscle fibers are shown by a red arrow, and fascia layer by a double-headed white arrow. Pgp9.5^+^/NFH^+^ nerves are shown with cyan arrows, and a Pgp9.5^–^/NFH^+^ nerve with a yellow arrow. ***D***, ***D’***, Expression of pgp9.5 (green) and NFH (red) in 11-year-old male marmoset masseteric muscle fibers. Objective is 10×. Pgp9.5^+^/NFH^+^ nerves are shown with cyan arrows, and Pgp9.5^+^/NFH^–^ nerves with white arrows. White horizontal bar shows 20-μm scale for each panel.

To further investigate this unexpected finding, we examined MM distribution of nerves with peptidergic CGRP^+^/NFH^–^ markers such as trpV1^+^, Nav1.8^+^, and pgp9.5^+^; and NFH^–^ markers such as trkC^+^ and PV^+^. Moreover, to evaluate whether these findings are translated across species, MM fiber types were labeled in the marmoset tissue. Pgp9.5 is marker for all types of sensory nerves, and could highlight location of non-peptidergic unmyelinated nerve fibers (Tokushige et al., 2006). Analysis 8–16 slides with MM sections from male mice showed that pgp9.5^+^ nerves innervating muscle fibers (Fig. 11*A*,*A’*), tendon of MM (Fig. 11*B*), and masseteric fascia (Fig. 11*C*) are always co-labeled by NFH (cyan arrows on all panels). Muscle fibers of male marmosets were predominantly innervated by pgp9.5^+^/NFH^+^ nerves (Fig. 11*D*,*D’*, cyan arrows). However, pgp9.5^+^/NFH^–^ nerves (Fig. 11*D*,*D’*, white arrows), which likely represent C-fibers, were seldomly detected on one of two MM sections observed with 10× objective. These findings imply that male mouse MM fibers, tendon and fascia are almost solely innervated by A-fiber myelinated fibers, while C-fiber unmyelinated fibers are rarely encountered in MM fibers of marmosets.

TRPV1 marks S3 and S4 groups of MM TG neurons containing peptidergic unmyelinated fibers. TRPV1^+^ nerves were not detected within masseteric muscle fibers (Fig. 11*A*,*A’*), tendon or fascia of MM (data not shown). However, some nerve branches of masseteric nerve located along muscle fibers contain TRPV1^+^ sensory fibers, which was also labeled by pgp9.5^+^ (Fig. 11*B*,*B’*, cyan arrows). Nav1.8^+^ fibers were highlighted by isolation of MM from Nav1.8-cre/Ai32-YFP mice. Electrophysiology recording showed that strong Nav1.8-YFP signal is detectable only in small neurons (S1–S4 groups), while M1 has weak Nav1.8 presence and S5 and M2–M4 have none. Hence, Nav1.8^+^ sensory fibers were not detected within muscle fibers and tendon. However, certain masseteric nerve branches contained Nav1.8^+^ fibers (Fig. 11*C*,*C’*, red arrow). Interestingly, we have detected multiple nerve branches adjusted or inside MM expressing both pgp9.5 and NFH, but not showing Nav1.8 (Fig. 11*C*,*C’*, yellow arrows). Expression patterns of CGRP^+^, pgp9.5^+^, Nav1.8^+^, and NFH^+^ nerves within MM indicate that muscle fibers, tendon and fascia are innervated by M1–M4 and maybe S5 TG MM neurons. In concordance with this, trkC, which is a marker for S5 and M2–M4 MM TG neurons (Fig. 13*C*), was detected in many NFH^+^ fibers (Fig. 11*D*,*D’*, cyan arrows). However, unlike trkC, anti-PV antibodies produced only weak labeling in masseteric nerve branches (data not shown). Overall, our data suggest that muscle fibers, tendon, and fascia of MM in mice and marmosets are almost exclusively innervated by S5 and M1–M4 nerves, which are mainly distributed in tendon and at junctions of superficial, middle, and deep parts of MM. Unmyelinated IB-4^+^, Nav1.8^+^, and TRPV1^+^ nerves (S1–S4 neurons) are in a subset of masseteric nerve branches, but do not terminate in muscle fibers, tendon, and fascia of mice MM (Fig. 13*A*,*B*). Additionally, it could not be excluded that WGA injected in MM could defuse into adjusted structures such as skin and especially, subcutaneous tissues, which contain unmyelinated C-fibers (see Discussion).

## Discussion

Mechanisms underlying masticatory myofascial pain are largely unknown. Masticatory myofascial pain involves MM in several conditions, including TMD and after some restorative surgeries in the head and neck. In this respect, understanding jaw muscle and particularly MM innervation is critical for dissecting cell-specific mechanisms controlling development of chronic masticatory myofascial pain. This understanding will eventually allow for the effective management of TMD pain (Dinsdale et al., 2021). In addition, masseteric nerve is affected during certain restorative surgeries and during transplantations/transfers, which are the most often performed cranial nerve manipulation technique used to treat patients with facial paralysis (Corcoran and Goldman, 2021). In this study, using backtracking from MM with WGA, reporter mice driven by promoters of well-characterized sensory neuronal markers, patch clamp electrophysiology and IHC, we identified and characterized types of TG sensory neurons innervating MM. This approach was successfully employed to identify and characterize neuronal groups in L3–L5 DRG and TG (Petruska et al., 2000; Xu et al., 2010; Patil et al., 2018). We clustered most recorded MM TG neurons into nine clusters (Fig. 13*C*; Table 2). One to two percent of recorded neurons did not fit these nine clusters. For example, MM TG neurons did not respond to ATP (Table 1), but from >350 analyzed neurons, seven responded to ATP.

There is not clear consensus on how the sensory neurons between DRG and TG differ. There is also not enough data on the biochemical differences between sensory neurons innervating the MM. Nevertheless, there is agreement that somatosensory neurons in different sensory ganglia have distinct anatomic, functional, physiological, and pathophysiological characteristics (Li, 2007; Belmonte and Viana, 2008; Gambeta et al., 2020). Thus, DRG are located in the intervertebral foramina at spinal levels that mainly innervate tissues within the trunk, legs and feet, while TG rest in Meckel’s cave and innervate the head and neck area (Vermeiren et al., 2020). Another anatomic distinction is proprioceptors with Aα-fibers are located in DRG but are outside TG in the mesencephalic TG nucleus (Jerge, 1963; Dubner, 1986). Since physiological function of tissues in the trunk, legs and feet are quite distinct from tissues in head and neck area, it is presumed that DRG and TG sensory neurons have distinct, specialized, and unique sets of proteins/mRNA. With the advent of RNA-seq, data from sorted sensory neurons and single-cells described some of these differences in recent years (Usoskin et al., 2015; Lopes et al., 2017; Nguyen et al., 2019; Zheng et al., 2019; Mecklenburg et al., 2020; Renthal et al., 2020). However, these sequencing data indicate that the differences between sensory neurons in DRG and TG are not dramatic and lack clear functional distinctions (Lopes et al., 2017; Mecklenburg et al., 2020; Sharma et al., 2020). These differences between DRG and TG neurons could be because of analysis of sensory neuronal profiles in whole ganglia, but not in specific subsets of sensory neurons innervating defined tissues (such as muscle, skin, tooth, dura, etc.). Considering that neuronal subsets may have distinct origins and may therefore be guided to different targets, innervating different tissues (Hockley et al., 2019), we focused our study on a specific subset of TG neurons innervating MM.

Ideally, our data need to be compared with DRG neurons innervating hind/fore limb muscle. However, this information is not available. Single-cell data generated by several independent studies imply that difference between neuronal groups innervating limb skin and muscle maybe not substantial (Usoskin et al., 2015; Sharma et al., 2020). Thus, our data indicate that 20–25% of L3–L5 DRG neurons were labeled by WGA injected into limb muscles (Mecklenburg et al., 2020). Single-cell sequencing of thousands of sensory neurons clustered major phenotypic differences of DRG groups innervating the skin and muscle, for example, proprioceptors were clustered as a separate group (Usoskin et al., 2015; Sharma et al., 2020). Taken all these points into consideration, we compared our results with published data from whole L3–L5 DRG. S1 and S2 groups, which are Nav1.8^+^, but not CGRP^+^, are classic CAP-unresponsive IB-4^+^ non-peptidergic sensory neurons (Stucky and Lewin, 1999). Unlike L3–L5 DRG neurons (Lou et al., 2015), MM TG IB-4^+^ neurons were not MrgprD^+^. Functional studies on DRG neurons distinguished two types of skin innervating IB-4^+^/MrgprD^+^ neurons: C-polymodal nociceptors (PMN) and C-mechano-nociceptors (C-MN; Liu et al., 2012; Lou et al., 2015). For MM TG neurons, it is not clear what functions could be assigned to S1 and TRPA1 containing S2 groups. S3 and S4 groups containing CGRP, TRPV1 and Nav1.8 are likely C-PMN-like with similarities to DRG neurons (Usoskin et al., 2015; Patil et al., 2018; Sharma et al., 2020). The last small-sized MM TG neuronal group characterized was S5. It had unique electrophysiological properties (Table 1) and we observed expression of CGRP and trkC, but not Nav1.8 in S5 group MM TG neurons. There is no analog for S5 neurons in L3–L5 DRG; and it is unknown whether S5 express NFH and belong to nociceptors or LTMR. Unlike DRG neurons innervating skin, MrgprA3^+^ (C-PMN; Han et al., 2013), CGRP^–^/TRPV1^+^ (also known as somatostatin^+^; Usoskin et al., 2015), and TH^+^ (also known as C-LTMR; Seal et al., 2009) groups were not found among MM TG neurons.

As it was previously reported (Sato et al., 2018), medium-sized neurons are most abundant among MM TG neurons (Fig. 13*C*). Moreover, in L3–L5 DRG innervating either skin or muscle, all CGRP^+^ neurons were found inside of the Nav1.8^+^ subset (Patil et al., 2018), while S5, M1, and M2 MM TG neuronal groups expressed CGRP but little-to-no Nav1.8 (Fig. 13*C*). M1 and M2 medium-sized MM TG peptidergic neurons produced large inward currents on application of 5HT. This property distinguished M1 and M2 from other MM TG neurons. M1 contains CGRP, a no-to-low level of Nav1.8, and no trkC. Considering previous research on DRG (Usoskin et al., 2015; Patil et al., 2018), M1 could be a peptidergic myelinated A-fiber nociceptor (Aδ-HTMR). However, it is unclear whether previously described A-fiber nociceptors are distinct in limb skin versus muscle (Usoskin et al., 2015; Sharma et al., 2020). L3–L5 DRG contains two types of Aδ-HTMR, which express CGRP, Nav1.8, but not trkC, can be distinguished by the presence of NPY2R marker (Patil et al., 2018; Sharma et al., 2020). In contrast, TG MM M2 neurons have CGRP, trkC, and a no-to-low level of Nav1.8. Additionally, NPY2R-tdTomato expression have not been detected in TG neurons (Wu et al., 2018). Such expression patterns in M2 MM TG neurons complicates their functional assignment. However, the presence of two A-fiber HTMR groups innervating MM has been reported (Wong et al., 2014); therefore, M2 could be a separate group of A-fiber HTMR, which are sometimes designated as Aβ-HTMR (Djouhri et al., 1998). MM is innervated by two TG neuronal groups, M3 and M4, that are not revealed in CGRP^cre-ER/+^ and Nav1.8^cre/+^ reporter mice. However, M3 and M4 have been recorded among WGA^+^/Nav1.8-cre^–^, WGA^+^/trkC-cre^+^, and WGA^+^/PV-cre^+^ TG neurons. In L3–L5 DRG innervating limb skin and muscle, functional studies suggested that trkC^+^/PV^+^ are either skin Aβ-LTMRs or muscle proprioceptors (Bai et al., 2015). Since, TG do not have proprioceptors (Jerge, 1963), it could be presumed that M3 and M4 may be MM TG Aβ-LTMRs neurons. Subgroup of skin Aβ-LTMRs, called Aβ-Field, express calbindin D28 (Calb) as a marker (Arcourt et al., 2017; Sharma et al., 2020). Since Calb is not in MM TG neurons, it could be speculated that M3 and M4 are likely rapidly adapting Aβ-LTMRs (Aβ-RA-LTMRs). Interestingly, electrophysiological properties of M3 is similar to DRG skin innervating Aβ-RA-LTMRs nerves, but M4 strongly resembles DRG muscle innervating proprioceptors (Patil et al., 2018). Thus, their AP, a unique fast AHP, unresponsiveness to algesics and their outward I parameters are almost identical (Fig. 4*B*,*D*; Table 1) versus DRG group M7 (Patil et al., 2018). Skin hairs are innervated by C-LTMR or/and Aδ-LTMR DRG neurons, which express TH and trkB markers, respectively (Seal et al., 2009; Rutlin et al., 2015; Usoskin et al., 2015). Amongst MM TG neurons these neuronal groups have not been recorded.

**Figure 12.**
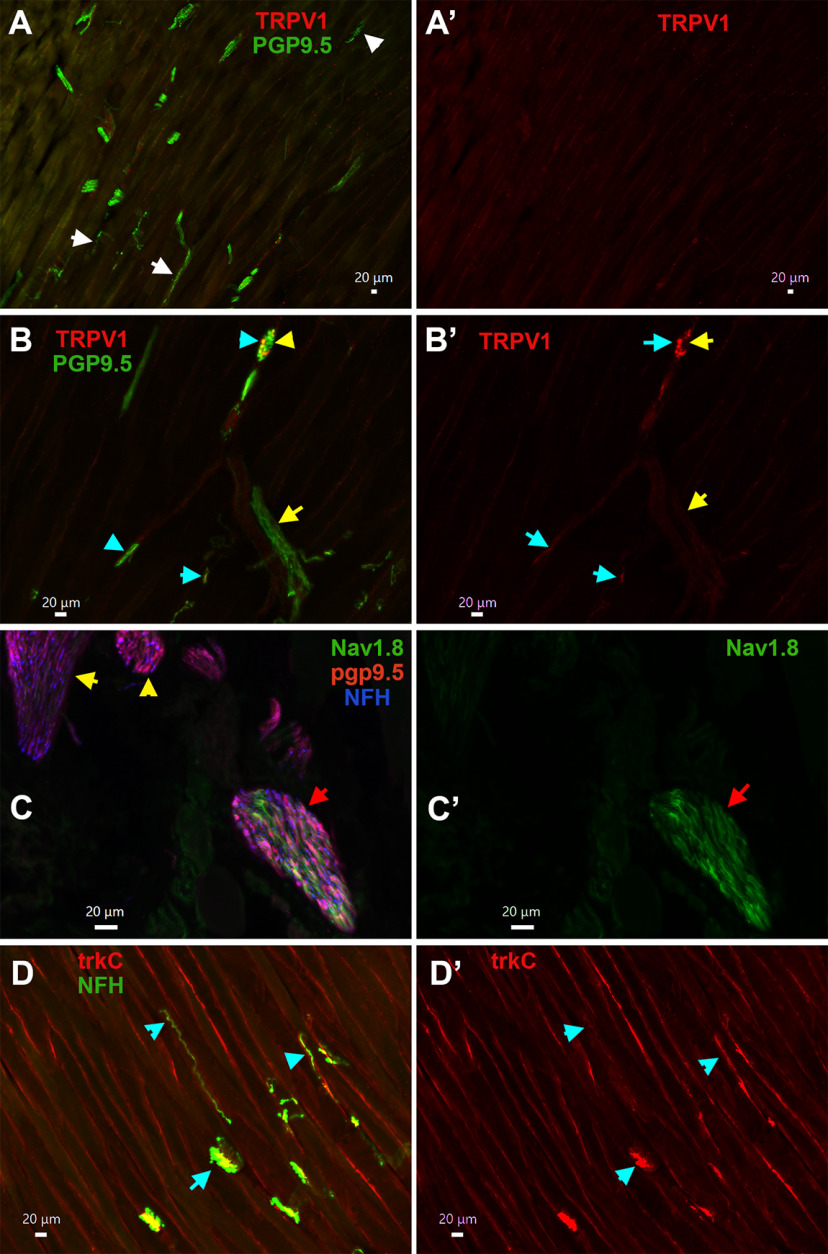
Expression of trpV1^+^, Nav1.8^+^, and trkC^+^ sensory neuronal afferent fibers in MM. ***A***, ***A’***, Expression of pgp9.5 (green) and TRPV1 (red) nerves in masseteric muscle fibers. Objective is 10×. TRPV1^–^/pgp9.5^+^ nerves are marked with white arrows. ***B***, ***B’***, Expression of pgp9.5 (green) and TRPV1 (red) nerves in nerve bundles located within masseteric muscle fibers. Objective is 20×. TRPV1^+^/pgp9.5^+^ nerves are marked with cyan arrows. Yellow arrows show nerve bundles. ***C***, ***C’***, Expression of Nav1.8-Ai32 (green), pgp9.5 (red), and NFH (blue) fibers in masseteric nerve branches. Objective is 40×. A red arrow shows a branch with Nav1.8-Ai32^+^ fibers, and yellow arrows mark Nav1.8-Ai32^+^ branches. ***D***, ***D’***, Expression of trkC (red) in NFH^+^ (green) nerves within masseteric muscle fibers. Objective is 20×. TrkC^+^/NFH^+^ nerves are marked with cyan arrows. White horizontal bar shows 20-μm scale for each panel.

Distribution of nerves in masseteric muscles has been studied in rats, rabbits, pig and cats (Gottlieb et al., 1984; Herring et al., 1989; Luo et al., 1991; Peker et al., 2001). It was shown that the masseteric nerve is subdivided into at least three to five subbranches within MM (Fig. 13*A*,*B*). Myelin sheets surrounding the unmyelinated nerve fibers disappeared as the fibers terminate (Herring et al., 1989). Our data shows that unmyelinated and myelinated fibers travel together in a main trunk of the masseteric nerve (Fig. 9*A*,*A’*). Similarly, nerve branches of the masseteric nerve contain either both unmyelinated and myelinated fibers or only myelinated fibers (Fig. 12*C*,*C’*). It was reported that distribution of different types of fibers occur throughout MM and are independent of fiber type (Herring et al., 1989). First, the data indicate that afferent fibers are concentrated in tendons and at junctions between deep, middle, and superficial parts of MM (Fig. 9*B*,*C*,*C’*). Second, importantly, male mouse muscle fibers, tendons, and masseteric fascia of MM were almost exclusively innervated by myelinated NFH^+^ afferent nerves (Figs. 9*B*,*C*,*C’*,*D*, 10, 11*A–C*). Likewise, male marmoset MM were predominantly innervated by myelinated NFH^+^ afferent nerves (Fig. 9*D*,*D’**).* Mouse MM TG neurons with myelinated fibers belong to M1–M4 groups, and maybe group S5 as well. IHC could detect unmyelinated MM TG neurons, which could be identified as Nav1.8^+^/NFH^–^, TRPV1^+^, and/or pgp9.5^+^/NFH^–^ afferent nerves, only within a subset of masseteric nerve branches (Fig. 12*A–D’*). It is not clear where these unmyelinated fibers terminate, since they were not detected by IHC in MM fibers, MM tendons and masseteric fascia. Accordingly, WGA injection into MM could label small TG neurons (S1–S4) found in the main trunk or sub-branches of the masseteric nerve, and/or adjacent to MM subcutaneous tissues and skin via WGA diffusion (Fig. 13*A*,*B*).

In conclusion, our data show that MM is almost exclusively innervated by myelinated nerves that can be divided into S5, M1–M4 neuronal groups (Fig. 13*A–C*). WGA injections into MM also revealed small sized TG neurons (S1–S4), nerve fibers from which are within the masseteric nerve or terminate in adjusted to MM structures (Fig. 13*B*,*C*). Properties of these MM TG neuronal groups have similarities and many distinctions from the well-described and characterized L3–L5 DRG neurons innervating limb skin and muscles (Petruska et al., 2000; Usoskin et al., 2015; Patil et al., 2018; Sharma et al., 2020). These differences could imply distinct functional consequences during naive and pathologic conditions affecting MM. It also suggests that TG MM neurons may contain a discrete subset of receptors and channels, which could uniquely regulate sensitization of MM TG neurons during masticatory myofascial pain. Finally, our novel report on the innervation of MM in a non-human primate species, the common marmoset, is important for many reasons. Because of the close relatedness of nonhuman primates and humans, delineation of the MM in the marmoset is important to support its use as a potential animal model MM-related pathology. Further, identification of similarities between marmoset and mouse MM further supports the use of both species as preclinical models for development of intervention approaches in this area.

## Synthesis

Reviewing Editor: Miriam Goodman, Stanford University Decisions are customarily a result of the Reviewing Editor and the peer reviewers coming together and discussing their recommendations until a consensus is reached. When revisions are invited, a fact-based synthesis statement explaining their decision and outlining what is needed to prepare a revision will be listed below. The following reviewer(s) agreed to reveal their identity: Joriene De Nooij.

Masseter sensory neurons - with their cell bodies in trigeminal ganglia - are identified though WGA back-tracing from the masseter muscle. Analyses of dissociated and cultured WGA-labeled neurons is linked to broadly defined TG sensory subclasses that are identified through the use of select genetic fluorescent reporters. Together these studies reveal nine physiologically and molecularly distinct masseter sensory subclasses, including peptidergic, non-peptidergic, and classes of low threshold mechanoreceptive afferents. Both Rev1 and Rev2 commented on the quality and quantity of the electrophysiological recordings supporting the classification of TG sensory neurons. Rev1 challenges the authors to increase the impact of the manuscript by addressing ineffective communication of data and the discussion of the results.

Below are specific opportunities for improvement noted by both reviewers

1. The authors fail to communicate the relevance of the work/results (or even the motivation for initiating these studies) in a broader context. There are some attempts at justifying the work (“MM sensory neurons are affected in mandibular disorders”) but there is almost never a consistent motivation as to why a physiological description of the many sensory subtypes that innervate the masseter is important in this context (the reasons seem to shift several times over). In addition, retrospectively, do the observed MM TG sensory subtypes make sense with the reported pathology of some of the disorders that have been mentioned?

2. A second major problem is the comparison to DRG subtypes. In several instances and in the conclusion (“In conclusion, our data show that MM is innervated by a unique subset of sensory neurons, which is substantially different from the well-described and characterized L3-L5 DRG neurons”), distinctions between MM TG neurons and DRG neurons are called out (e.g. a lack of ATP responsive neurons, or MrgprA3+ neurons) but this is in a sense comparing apples and pears. A more relevant comparison would be between MM TG and back filled hind/fore limb muscle DRG neurons since muscle innervating neurons may be under sampled in recordings of L3-L5 DRG neurons (many of which innervate skin). In reality the difference between MM TG and DRG sensory neurons is expected (based on the intro - “There is consensus that somatosensory neurons in different sensory ganglia have distinct anatomical, neurochemical, functional, physiological, and pathophysiological characteristics.”) and should perhaps be noted primarily in the introduction. This prior knowledge is overlooked elsewhere in the manuscript in which the authors state that this work “provides the first demonstration that MM innervating TG neurons are substantially distinct from DRG neurons.” It cannot be both previous understood and newly discovered.

3. Recognizing that these sensory neurons are likely to be mechanosensitive and that analyzing mechanically activated currents is a significant undertaking in and of itself, it would helpful to comment on how this work lays the ground work for future characterization of their mechanically activated currents.

3. The authors identify nine different MM innervating TG subtypes but there is no estimation as to whether this is presumed to be all subtypes or if there may be others that remain undetected by WGA labeling (do all neurons label equally efficiently with WGA?; did the authors also try other tracers to compare labeling efficiency?) or due to the isolation method (too sensitive to survive).

4. Some of the reporter lines that are used in the study rely on tamoxifen-dependent activation of the Cre-driver, yet others do not have this temporal control. Many adult sensory neuron subtype molecules are often transiently expressed in a broader neuronal subset during early development. To what extent could this impact the interpretation of the current studies?

5. The MrgD antibody could use further validation with in situ analysis.

6. Considering there is a lot of attention for the muscle topography and an attempt to link distribution of MM TG subclasses to certain muscle regions, the manuscript could benefit of a schematic diagram of the MM and its innervation pattern.

7. The images in Figure 6 (Figures are not numbered) are uninterpretable without high power insets.

8. Overall the writing lacks precision, making data/explanations difficult to understand (e.g. line 486-487: “It could be the masseter fascia or adjusted to MM structures, since WGA injection could label fibers innervating the masseter fascia and fibers within nerve branches contacting MM.”)

10. Footnote #11 in Table 1: “11 - Some (≈15%) M3 neurons respond to 5HT (30 um)” What does 30 um refer to?

11. Were liquid junction potential corrections made in Erest calculations? Please state whether or not this correction was made.

12. Line 13 “dis not have” typo

## Author Response

We would like to thank each Reviewer and the Reviewing Editor for their valuable critiques, and expertise provided in the review of our manuscript. We acknowledge each critique and have provided new data (additional figures) and revised the manuscript (revisions in red font) accordingly. Below, we list each Reviewer's and Editors' comments in bold followed by our response in regular font.

Significance Statement Comments for Author (Required):

The significance statement makes a claim about novelty that is distracting - “To our knowledge this provides the first demonstration that MM innervating TG neurons are substantially distinct from DRG neurons.” Please remove. Please also provide a sentence telling the reader about the implications of having a better understanding of the sensory innervation of the masseter muscle.

We removed the statement about novelty from “Significance Statement” and provided a sentence at the beginning about the implications of having a better understanding of the sensory innervation of the masseter muscle.

Comments on the Visual Abstract for Author (Required):

The revised manuscript includes an additional three-panel figure (Figure 13) that provides a visual abstract of our findings. We also moved the panel 4E into this new figure.

Synthesis Statement for Author (Required):

Masseter sensory neurons - with their cell bodies in trigeminal ganglia - are identified though WGA back-tracing from the masseter muscle. Analyses of dissociated and cultured WGA-labeled neurons is linked to broadly defined TG sensory subclasses that are identified through the use of select genetic fluorescent reporters. Together these studies reveal nine physiologically and molecularly distinct masseter sensory subclasses, including peptidergic, non-peptidergic, and classes of low threshold mechanoreceptive afferents. Both Rev1 and Rev2 commented on the quality and quantity of the electrophysiological recordings supporting the classification of TG sensory neurons. Rev1 challenges the authors to increase the impact of the manuscript by addressing ineffective communication of data and the discussion of the results.

We thank the Reviewers and the Editor for summarizing the approaches and findings in the manuscript. We agree with Reviewer #1; accordingly, we have revised the manuscript; and have included additional data. Responses to specific critiques are below.

1. The authors fail to communicate the relevance of the work/results (or even the motivation for initiating these studies) in a broader context. There are some attempts at justifying the work (“MM sensory neurons are affected in mandibular disorders”) but there is almost never a consistent motivation as to why a physiological description of the many sensory subtypes that innervate the masseter is important in this context (the reasons seem to shift several times over). In addition, retrospectively, do the observed MM TG sensory subtypes make sense with the reported pathology of some of the disorders that have been mentioned?

We agree that the rationale was not presented well; and accordingly, “Introduction” was substantially rewritten and revised. Briefly, to better understand TMD pain mechanisms and sensitization of sensory neurons, we need to know what types of sensory neurons innervate the masseter muscle. Similarly, surgeries that lead to muscle (including the masseter muscle) damage are particularly painful. Reconstruction surgeries involving the masseter muscle are performed often, and for effective non-opioid post-op pain control, sensory neurons innervating the MM need identification and further study. Unfortunately, neurons innervating the MM have not been studied at a sufficient level to precisely explain (and even correlate) the reported pathology found during masticatory myofascial pain.

2. A second major problem is the comparison to DRG subtypes. In several instances and in the conclusion (“In conclusion, our data show that MM is innervated by a unique subset of sensory neurons, which is substantially different from the well-described and characterized L3-L5 DRG neurons”), distinctions between MM TG neurons and DRG neurons are called out (e.g. a lack of ATP responsive neurons, or MrgprA3+ neurons) but this is in a sense comparing apples and pears. A more relevant comparison would be between MM TG and back filled hind/fore limb muscle DRG neurons since muscle innervating neurons may be under sampled in recordings of L3-L5 DRG neurons (many of which innervate skin). In reality the difference between MM TG and DRG sensory neurons is expected (based on the intro - “There is consensus that somatosensory neurons in different sensory ganglia have distinct anatomical, neurochemical, functional, physiological, and pathophysiological characteristics.”) and should perhaps be noted primarily in the introduction. This prior knowledge is overlooked elsewhere in the manuscript in which the authors state that this work “provides the first demonstration that MM innervating TG neurons are substantially distinct from DRG neurons.” It cannot be both previous understood and newly discovered.

There are several crucial points here. Unfortunately, detailed information (i.e. single-cell sequencing) on DRG neurons innervating hind/fore limb muscle is not available and so we are unable to compare to MM TG. Although we expect differences “between MM TG and DRG sensory neurons", it is unknown how big these differences are. Importantly, based on some publications (PMID: 31915380; PMID: 29018326), a substantial number of researchers think that there is no principial difference between TG and DRG neurons (see “Discussion”). Critical differences have been presumed but have never been shown. However, we agree that more information on these distinctions should be obtained by different approaches, especially single cell seq from labeled neurons (this is not a simple task) and manipulation of neuronal groups with regards to their mechno-gated responses and functional changes during pain conditions. These types of studies will need the baseline information that is presented in this manuscript. Overall, we revised “Discussion” and included these points, and stipulated what types of DRG neurons (innervation-wise) have been compared to MM TG neurons.

With regards to the issue of under sampling of muscle innervating DRG neurons, our experience (PMID: 32943709) showed that thigh muscle injected with WGA produces 25% of labeled DRG neuron numbers versus 55-65% intraplanar injected WGA. Single-cell sequencing information generated using the Genomic X-10 platform, which gets information from thousands of sensory neurons, will not miss clustering of DRG L3-L5 neurons innervating muscle. Thus, all published single-cell seq on DRG L3-L5 precisely show proprioceptor neuronal groups, which only innervate hind/fore limb muscle. Nevertheless, similarities/differences between DRG L3-L5 C and A-fiber neuronal groups innervating skin and muscle are unknown. Problem is that clustering analysis could still miss differences if they are “marginal”. Definition of “marginal” is very loose here since clustering analysis has some inherent disconnection between mathematic and biological meaning. Thus, different criteria during analysis could produce different clustering outcomes.

3. Recognizing that these sensory neurons are likely to be mechanosensitive and that analyzing mechanically activated currents is a significant undertaking in and of itself, it would helpful to comment on how this work lays the ground work for future characterization of their mechanically activated currents.

We included this critical topic in the “Discussion” section of the revised manuscript. Mechanical currents of these neuronal subtypes will definitely be analyzed in the future. However, such work will be preceded by single-cell sequencing, which is quite challenging for “small” (relatively small) subsets of neurons. This challenge is due to difficulties and it may be impossible to use automated the Genomic X-10 platform in such single-cell seq experiments.

4. The authors identify nine different MM innervating TG subtypes but there is no estimation as to whether this is presumed to be all subtypes or if there may be others that remain undetected by WGA labeling (do all neurons label equally efficiently with WGA?; did the authors also try other tracers to compare labeling efficiency?) or due to the isolation method (too sensitive to survive).

Great point. There is a nonuniform labeling by WGA for different subpopulations of DRG neurons and it may not label all innervating neurons (see PMID: 2351563). Thus, most of the small cells were heavily stained, whereas the large cells showed less or maybe occasionally no staining. Could it contribute to missing entire sub-group of MM innervating neurons? The probability is not zero. However, in our opinion, it is unlikely since every neuronal group had large variation in neuronal sizes. Moreover, TG seldomly have neurons with diameter >60 m, which is often encountered in DRG neurons. We included these thoughts in the “Result” section.

5. Some of the reporter lines that are used in the study rely on tamoxifen-dependent activation of the Cre-driver, yet others do not have this temporal control. Many adult sensory neuron subtype molecules are often transiently expressed in a broader neuronal subset during early development. To what extent could this impact the interpretation of the current studies?

This is an important point. In a previous publication (PMID: 29864146), we identified reporters that are expressed in broader neuronal subsets during early development. These reporters are vGlut3-cre/tdTomato and TRPV1-cre/tdTomato, while PV-cre and Nav1.8-cre correctly represent expressions of PV and functional Nav1.8 in adult mice. TRPV1-GFP and Htr3a-GFP are conventional transgenic reporters. Expression patterns of GFP are dictated by trpv1 or the Htr3a promoter and change with age and conditions. Overall, we have chosen reporters that are not impacted by early developmental changes. Otherwise, we agree interpretation of results could be compromised.

6. The MrgD antibody could use further validation with in situ analysis.

This is a great point. However, in situ approach is not set up in our lab. Our research have shown that it will take enormous cost (thousands) to set this method and perform RNA-scope. Hence, we opted for an alternative method. Inducible MrgprD-cre-ER/tdTomato reporter was used for back-tracing with WGA-488 from masseter muscle to find out whether MM innervating neurons express MrgprD (see new Figure 6). Results are almost identical to those with MrgprD antibodies.

7. Considering there is a lot of attention for the muscle topography and an attempt to link distribution of MM TG subclasses to certain muscle regions, the manuscript could benefit of a schematic diagram of the MM and its innervation pattern.

We totally agree with this. Schematic (last figure; Fig 13) was made with help of multi-media professionals. This schematic shows masseter muscle topography, position of adjunct structures to MM and innervation of MM by masseteric nerve and its branches.

8. The images in Figure 6 (Figures are not numbered) are uninterpretable without high power insets.

This is a bit confusing. Figure 6 is taken with x20 objective. Maybe it has too many panels and becomes uninterpretable? Maybe Figure 7C is uninterpretable since it has indeed low magnification (x2)? First, we have numbered all figures. Additionally, we have made the following changes: split Figure 5 and 6 into two figures (each), added a high-power insert (separate capture with x20 objective) for Figure 7C (now it is number 9), added fascia image to the new Figure 9, split Figure 8 (now they are Figures 10 and 11), and added IHC from masseter muscle of primates to new Figure 11 (see point 9).

9. Overall the writing lacks precision, making data/explanations difficult to understand (e.g. line 486-487: “It could be the masseter fascia or adjusted to MM structures, since WGA injection could label fibers innervating the masseter fascia and fibers within nerve branches contacting MM.”)

We agree that this is an important point; and was not optimally presented due to lack of data. We have decided to generate additional data for addressing some of these questions. First, we investigated what fiber types are found in masseter muscle fascia. We found only NFH+/pgp9.5+ (i.e. A-fibers) there. So, it appears that male mouse masseter muscle is exclusively innervated by A-fibers. Next, we looked whether this was replicated in primates. IHC on masseter muscle of marmosets (11 and 15 years old) was performed. In primates, a similar pattern was observed, with the bulk majority of nerves are A-fibers with a few (≈1-2%) presenting as pgp9.5+/NFH- fibers (i.e. C-fibers). We included these data in the revised manuscript. Overall, masseter muscle is almost exclusively innervated by A-fibers. WGA-labeled C-fibers are probably due to diffusion of dye into adjacent MM structures. This part of discussion was revised. Schematic of masseter muscle innervation was included in the revised manuscript. It also helps to shows this possibility of WGA diffusion.

10. Footnote #11 in Table 1: “11 - Some (≈15%) M3 neurons respond to 5HT (30 um)” What does 30 um refer to?

Thanks for noting this. It is a typo. It should be 30 uM (concentration of 5HT).

11. Were liquid junction potential corrections made in Erest calculations? Please state whether or not this correction was made.

Nernst-Planck or Henderson equation was not used for calculating liquid junction potential (LJP). We also did not use Vmeter = Vcell + LJP equation for correction of Erest. We have stated this in the “Material and Method” section of the revised manuscript. LJP was not corrected, since no gluconate, aspartate or methanesulfonate was used in the pipette solution. Usually, low mobility of these large anions results in accumulation in the pipette and produces a significant LJP. Additionally, 3-4 min after patch was made, cells were allowed to establish equilibrium in whole-cell configuration. Overall, we think the external and internal solutions and procedure used produced a low LJP.

12. Line 13 “dis not have” has a typo

Thank you for noting this. This part of the text was substantially revised.
